# Cytological maps of lampbrush chromosomes of European water frogs (*Pelophylax esculentus* complex) from the Eastern Ukraine

**DOI:** 10.1186/1471-2156-14-26

**Published:** 2013-04-16

**Authors:** Dmitry Dedukh, Glib Mazepa, Dmitry Shabanov, Juriy Rosanov, Spartak Litvinchuk, Leo Borkin, Alsu Saifitdinova, Alla Krasikova

**Affiliations:** 1 Saint-Petersburg State University, Oranienbaumskoie sch. 2, Stary Peterhof, Saint-Petersburg 198504, Russia; 2 Department of Ecology and Genetic, Population Biology and Conservation Biology, Evolutionary Biology Centre, Uppsala University, EBC Norbyvägen 18 D, Uppsala 75236, Sweden; 3 V.N. Karazin Kharkiv National University, Svobody Sq. 4, Kharkiv 61022, Ukraine; 4 Institute of Cytology Russian Academy of Sciences, Tikhoretsky pr. 4, St. Petersburg 194064, Russia; 5 Zoological Institute, Russian Academy of Sciences, Universitetskaia nab.1, St. Petersburg 199034, Russia

**Keywords:** Centromere, Chromosome, European water frog, Hybridization, Karyotype, Non-coding RNA, Nuclear body, Oocyte, Telomere

## Abstract

**Background:**

Hybridogenesis (hemiclonal inheritance) is a kind of clonal reproduction in which hybrids between parental species are reproduced by crossing with one of the parental species. European water frogs (*Pelophylax esculentus* complex) represent an appropriate model for studying interspecies hybridization, processes of hemiclonal inheritance and polyploidization. *P. esculentus* complex consists of two parental species, *P. ridibundus* (the lake frog) and *P. lessonae* (the pool frog), and their hybridogenetic hybrid – *P. esculentus* (the edible frog)*.* Parental and hybrid frogs can reproduce syntopically and form hemiclonal population systems. For studying mechanisms underlying the maintenance of water frog population systems it is required to characterize the karyotypes transmitted in gametes of parental and different hybrid animals of both sexes.

**Results:**

In order to obtain an instrument for characterization of oocyte karyotypes in hybrid female frogs, we constructed cytological maps of lampbrush chromosomes from oocytes of both parental species originating in Eastern Ukraine. We further identified certain molecular components of chromosomal marker structures and mapped coilin-rich spheres and granules, chromosome associated nucleoli and special loops accumulating splicing factors. We recorded the dissimilarities between *P. ridibundus* and *P. lessonae* lampbrush chromosomes in the length of orthologous chromosomes, number and location of marker structures and interstitial (TTAGGG)_n_-repeat sites as well as activity of nucleolus organizer*.* Satellite repeat RrS1 was mapped in centromere regions of lampbrush chromosomes of the both species. Additionally, we discovered transcripts of RrS1 repeat in oocytes of *P. ridibundus* and *P. lessonae*. Moreover, G-rich transcripts of telomere repeat were revealed in association with terminal regions of *P. ridibundus* and *P. lessonae* lampbrush chromosomes.

**Conclusions:**

The constructed cytological maps of lampbrush chromosomes of *P. ridibundus* and *P. lessonae* provide basis to define the type of genome transmitted within individual oocytes of *P. esculentus* females with different ploidy and from various population systems.

## Background

Interspecies hybridization is spread rather widely across different groups of living organisms though offspring of such mating are often sterile. Hybrid sterility reduces the exchange of genes between two species ensuring species divergence [1]. Nevertheless hybridization produces new gene combinations making hybrids more successful in evolution. Resolving the problems of fertilization and reproductive isolation from parental species can lead to appearance of new species in prospect [2-5]. Reproducibility of the majority of natural interspecies hybrids in vertebrates can be achieved in clonal ways of reproduction which are often accompanied by polyploidy: parthenogenesis (occurring in some fishes, lizards, and snakes), gynogenesis (ambystomes and some fishes), and hemiclonal inheritance (frogs, toads and some fishes).

The European water frogs of the *Pelophylax esculentus* complex represent an appropriate model for studying interspecies hybridization accompanied by hemiclonal inheritance and polyploidization (reviewed in [6]). This complex consists of two parental species – the lake frog *Pelophylax ridibundus* (genome composition RR) and the pool frog *P. lessonae* (LL), as well as natural hybridogenetic form – the edible frog *P. esculentus* (LR), the latter arising as a result of hybridization between the two parental species [7]. In the generations of hybrid frogs, the phenomenon of hemiclonal inheritance was registered: one of two genomes is eliminated from the germline, while the other (clonal genome) can be transmitted to gametes without recombination [6,8]. If hybrid males and females transmit identical clonal genomes, crossing between the two hybrids results in appearance of corresponding parental species. Parental individuals appearing in such a way often have developmental deviations and die before maturity (reviewed in [6]). Such problems in individual development of parental animals can be explained by accumulation of negative recessive mutations, which can not be removed from the clonal genome due to lack of recombination [6]. All these forms compose hemiclonal population systems, where *P. esculentus* transmit either L or R genome as a clonal one. The type of transmitted genome is correlated with parental species syntopic with hybrid frogs. In population systems, hybrid frogs can be represented not only by diploid animals but also by triploids with genotypes LLR and RRL. These latter forms most likely appear as a result of fertilization of egg cell with two sets of chromosomes by haploid sperm or vise versa [9].

Although hybridogenetic diploid frogs (*P. esculentus*) are widely known across temperate Europe from France in the west to Volga River in the east [6,10], natural polyploidy has been found in population systems distributed in western and central parts of Europe only [6]. The Seversky Donets River basin (Eastern Ukraine) is also inhabited by polyploids of *P. esculentus*[11]. The local population systems of water frogs include *P. ridibundus*, diploid *P. esculentus*, two forms of triploid *P. esculentus*, and even rare tetraploid *P. esculentus* (with LLRR genotype). Diploid hybridogenetic males in this region transmit clonally *P. lessonae* or *P. ridibundus* genomes, or both genomes in different gametes [11]. In some population systems of the basin, triploid hybrid frogs reached the majority of individuals. At the same time, water frog population systems found in the Seversky Donets River basin have some distinctive features from other hybrid formation centers. The first one is the isolation of the Seversky Donets River basin population system from European centers that produce triploid hybrid frogs approximately to the distances of 1000 km (eastern Poland) and 1500 km (western Hungary) [11]. The second one is the reproduction of hybrid frogs without *P. lessonae* since only several immature individuals were found in some local population systems [11-13]. In our present and future studies, the Seversky Donets River basin was chosen as main center for understanding the mechanisms of interspecies hybridization in European water frogs.

For studying mechanisms underlying the maintenance and dynamics of water frog populations, a cytogenetic analysis of karyotypes transmitted in gametes of parental and various hybrid animals of both sexes is required. The number of chromosomes in spermatocytes of *P. esculentus* can be estimated by examination of squash testis preparations and in drop preparations. Nevertheless, the main problem of definition the parental chromosomes in hybrid gametes is identical number and morphological resemblance of orthologous chromosomes in karyotypes of parental species [14-17]. DNA-flow cytometry approach resolves this problem and was successfully applied to identify the genome composition of male gametes in both parental species and hybrid frogs of *P. esculentus* complex from various population systems of the Eastern Ukraine [12]. However, genome composition in female gametes of water frogs from this region has never been previously determined. The original approach, which allows to estimate the chromosomal number in growing oocytes and to define the species-specific features of chromosomal morphology, is examination of giant lampbrush chromosomes microsurgically isolated from oocyte nucleus. This method was suggested and widely used in the pioneering studies of amphibian oocyte karyotypes (for a review, see [18,19]).

Lampbrush chromosomes are a form of meiotic chromosomes occurring in growing oocytes of many animals during the long diplotene period of prophase I of meiosis. Lampbrush chromosomes exist as highly extended half-bivalents with homologous chromosomes connected by chiasmata. They are characterized by distinctive chromomere-loop structure, and comprise conspicuous lateral loops, corresponding to trancriptionally active regions, and chromomeres that consist of inactive chromatin segments [18,20-23].

For the first time lampbrush chromosomes of the *Pelophylax esculentus* complex were described in 1979 [24], but only a decade later Bucci et al. [16] characterized lampbrush chromosomes of *P. ridibundus* and *P. lessonae* from Poland in detail. They also suggested that described lampbrush karyoptypes can be used for genome identification in oocytes derived from hybrid frogs, *P. esculentus*[16,25]. Lampbrush chromosomes of European water frogs are quite long (up to 500 μm in length), which correlates with the average size of their genomes (14.0–16.4 pg for a diploid genome; [11,26]). Like lampbrush chromosomes of many other amphibian and avian species, lampbrush chromosomes of European water frogs bear a variety of marker structures including loops with unusual morphology and complex organization, associated spherical bodies, and nucleoli, which altogether allow the identification of all individual chromosomes [16].

In this paper we perform a detailed analysis of oocyte karyotypes of *P. ridibunda* and *P. lessonae* originating from Eastern Ukraine and present comprehensive cytological maps of all parental lampbrush chromosomes of the European water frog complex, describing intraspecific variation between frogs from Ukrainian and Polish populations. We also characterize molecular composition of marker structures that distinguish orthologous lampbrush chromosomes of the parental species. Furthermore, we provide evidences for transcription of tandem repeats in centromere and telomere regions of chromosomes during the lampbrush stage of oogenesis in European water frogs.

## Results

**Figure 1:**
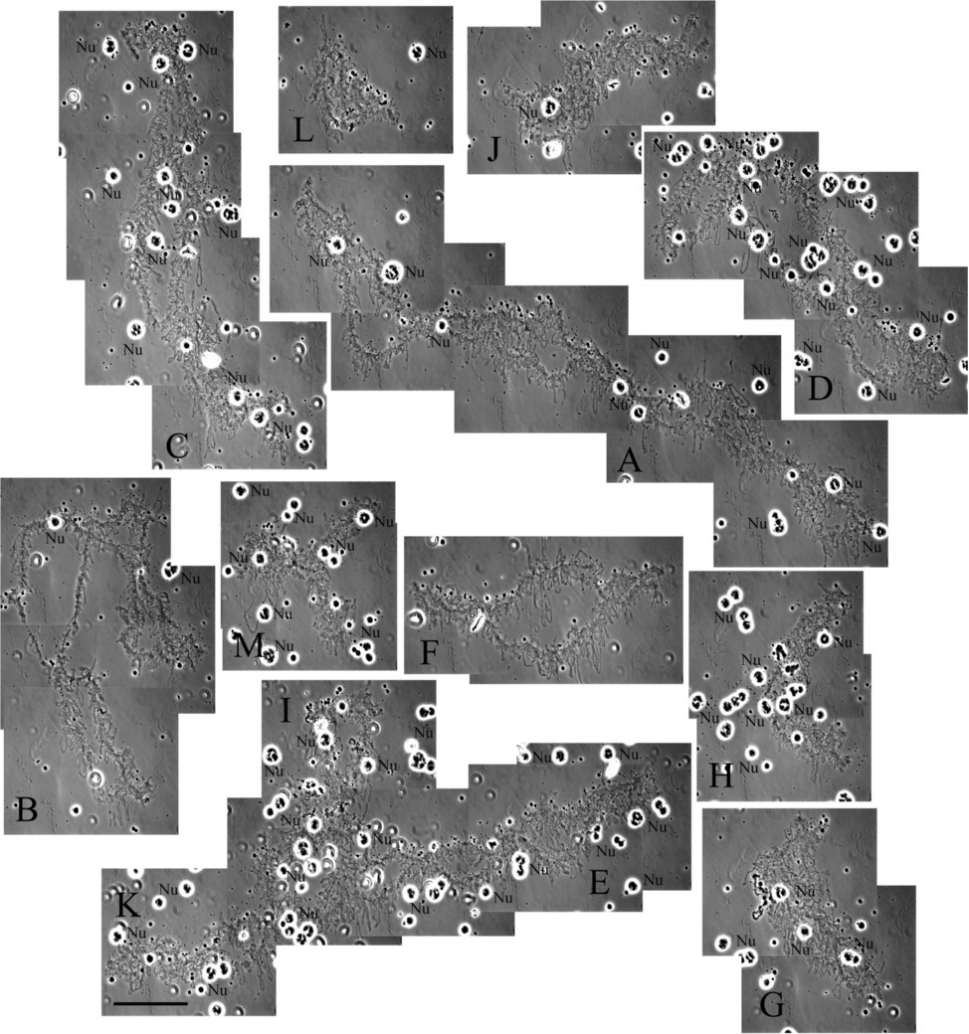
**Full set of lampbrush chromosomes from *P. ridibundus *(the lake frog) oocyte.** Oocyte chromosome set consists of 13 lampbrush stage bivalents with 5 large lampbrush chromosomes and 8 lampbrush chromosomes of smaller size. Lampbrush chromosomes were lettered according to their comparative size. Phase contrast micrograph. Nu – extrachromosomal and chromosome associated nucleoli. Scale bar = 50 μm.

In this work we first aimed to determine morphologically distinctive marker structures on lampbrush chromosomes of two parental species (the lake frog and the pool frog) of the European water frogs originating from Eastern Ukraine. For that purpose we analyzed and statistically treated data for 11 full sets of lampbrush chromosomes from *P. ridibundus* females and 10 preparations with full sets of lampbrush bivalents from *P. lessonae* females. Additional preparations of nuclear contents from 7 *P. ridibundus* oocytes and 6 *P. lessonae* oocytes were used for FISH and immunofluorescent staining procedures. The species assignment of all individuals was performed by genome size measurement using DNA flow cytometry. Individuals with C-values between 15.99 and 16.22 were referred to *P. ridibundus* species and individuals with C-values between 14.01 and 14.15 were considered as *P. lessonae* species according to Borkin and coauthors [11]. All oocytes examined had normal chromosomal number of 13 bivalents in each set. 5 large and 8 small bivalents were discerned in *P. ridibundus* and *P. lessonae* lampbrush karyotypes (Figure 1 and Additional file 1: Figure S1).

The relative chromosome sizes (chromosomal length relatively the longest one) of all lampbrush chromosomes in each karyotype were estimated (Tables 1 and 2), that allowed to define them by letters from A to M in the following description (Figure 1 and Additional file 1: Figure S1). For each chromosome of the both species the centromere index (ratio of the short arm length to the total chromosome length) was calculated (Tables 1 and 2). The estimated average number of chiasmata in a set of bivalents from lampbrush stage oocytes was 51.94 ± 1.37 for *P. lessonae* (n = 16) and 59.83 ± 1.73 for *P. ridibundus* (n = 18). Chiasmata frequency of *P. ridibundus* significantly differed from chiasmata frequency of *P. lessonae* (Student’s t-test, p<0.001).

**Table 1:** **Relative length and positions of marker structures on lampbrush chromosomes of *P. ridibundus***

**№**	**Relative length**	**Centromere index**	**Long marker loops**	**Lumpy loops**	**Giant structure**	**Nucleolus**	**Spheres**	**Large granules**
A	100	43.5	2; 37; 99					78
B	87.1	30.88	19.7; 41.1	25.49			97.5	
C	70	29.71	77.57	83.29				
D	62.1	22.12	0; 31.88	29.31				20.29
E	56	41.61		34.64				
F	43.6	38.53	53.9					
G	38.5	44.16	11.69; 55.58; 67.53					
H	35.8	32.68	56.7; 69.27		39.11	62.29		
I	31.4	40.76	32.8; 62.78; 72.61					
J	29.9	10.7	51.84	33.44; 64.88				
K	29.2	15.41	21.58	34.93			11.3	
L	23.4	27.78	44.02	60.68				
M	20.1	27.36	41.29					

**Table 2:** **Relative length and positions of marker structures on lampbrush chromosomes of *P. lessonae***

**№**	**Relative length**	**Centromere index**	**Long marker loops**	**Lumpy loops**	**Giant structure**	**Nucleolus**	**Spheres**	**Large granules**
A	100	39.1	1.1; 15.8; 97.5					88.4; 98.1
B	83.8	34.37	54.3; 84.61; 93	37.95			96.78	
C	75.9	37.9	19.5; 35.44; 69.57	43.35				0; 82.74
D	68.4	23.68	72.95	29.39; 85.09				75.73
E	61	41.31	0; 19.18	30.16; 45.57; 48.52				0
F	45	39.6	58.44	35.33				
G	40.1	41.9		36.41; 53.12; 71.82				
H	35.7	36.69	59.94	47.9				
I	34.5	7.3	50.72					
J	33.4	16.47	0; 23.35	39.52			13.77	
K	28.1	23.13		38.43				
L	26	45.77	83.08	66.92			32.69	
M	21.9	26.48	47.03	35.16; 67.12				

Morphological analysis revealed following regularly identifiable longitudinal landmarks on lampbrush chromosomes: centromeres, terminal and interstitial granules, complex lumpy loops with dense RNP-matrix, long marker loops, giant loops with dense RNP-matrix, and chromosome associated spheres and nucleoli (Tables 1 and 2). It should be stressed that during the lampbrush chromosome stage of oogenesis, thousands of extrachromosomal bodies, such as amplified nucleoli, spheres, B-snurposomes, appear in the nucleus of growing amphibian oocyte and can be observed on spread preparations (Figure 1) [22].

**Figure 2:**
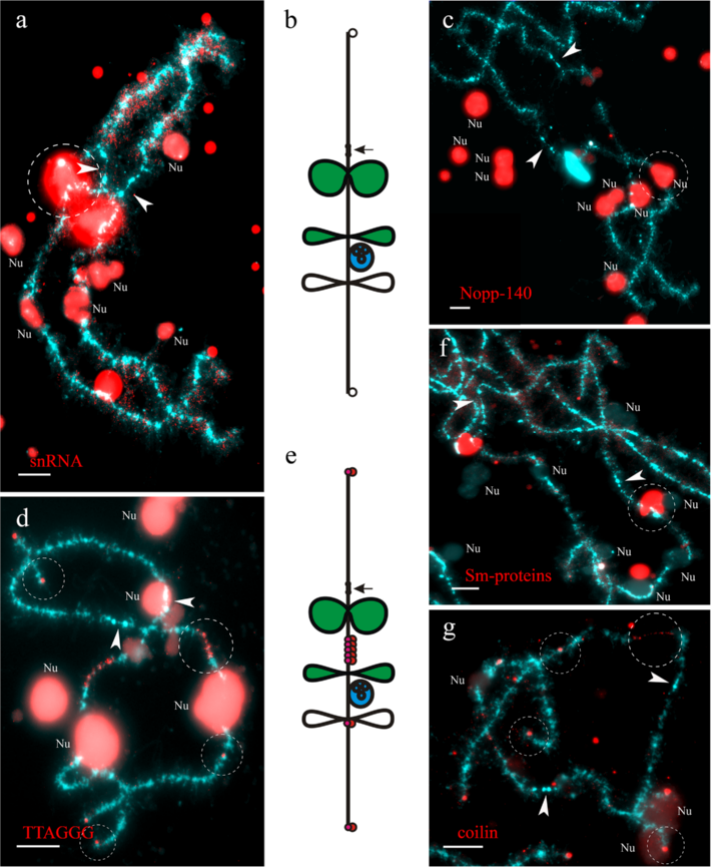
**Immunodetection of components of landmark structures and FISH mapping of (TTAGGG)-repeat sites on lampbrush chromosome H of *P. ridibundus *(the lake frog). a, f**. Identification of giant structures (giant fusing loops) and marker loops enriched with splicing factors. Immunofluorescent staining with antibodies K121 against TMG-cap of snRNA (**a**) and antibodies Y12 against Sm-proteins (**f**). **c**. Identification of chromosome associated nucleolus using immunofluorescent staining with antibodies No-185 against nucleolus protein Nopp-140. Dotted lines indicate marker structures. **d**. FISH mapping of (TTAGGG)_n_ repeat on lampbrush chromosome H. Telomeres and interstitial (TTAGGG)-repeat sites were detected and shown by dotted lines. **g**. Detection of coilin-positive granules (indicated by dotted lines) by immunofluorescent staining with R288 antibody. Arrowheads show centromeres. Nu – extrachromosomal nucleoli. Chromosomes are counterstained with DAPI. Scale bars = 10 μm. **b, e**. Color indication of marker structures according to their molecular components on cytological map of lampbrush chromosome H. Loops accumulating splicing factors are colored green, chromosome associated nucleolus – blue, coilin accumulating granules – red.

Immunofluorescent staining allowed to sort marker structures that have distinct morphological appearance and to identify additional marker loops on lampbrush chromosomes of both species. Antibodies К121 against 2,2,7-trimethylguanosine (TMG) cap of most of the small nuclear RNAs (snRNAs) and mAb Y12 against symmetrical dimethylarginine allowed to identify marker loops, accumulating spliceosomal components (Figures 2a, f, Additional file 1: Figure S1). Example of lumpy loops enriched with snRNPs is shown on Figure 2f. These mAbs also stained extrachromosomal nuclear organelles (Figure 2a, Additional file 1: Figure S1), which in *Xenopus* oocytes were referred as B-snurposomes and spheres [23]. Immunostaining with antibodies against nucleolar proteins Nopp-140, No38 and fibrillarin was aimed to detect nucleoli associated with lampbrush chromosomes (Figure 2c). It is important to note that these proteins also concentrate in multiple extrachromosomal nucleoli (Figure 2c). Antibodies against coilin were applied to nuclear contents preparations in order to reveal coilin-positive bodies and chromosome associated granules (Figure 2g).

### Construction of cytological lampbrush chromosome maps

Detailed cytological maps of lampbrush chromosomes of two water frog species were constructed on the basis of statistical treatment of data from lampbrush chromosome micrographs. On these maps, relative positions of various marker structures were plotted. For example, lampbrush chromosome H (LBC H) of *P. ridibundus* bears all specified marker structures except for spheres (Table 1 and Additional file 2: Figure S2b), which were pointed out on corresponding cytological map of this lampbrush chromosome (Additional file 2: Figure S2a). It is worth mentioning that the average size of giant loops on LBC H was estimated as 12 μm; usually giant loops formed by sister chromatids were found fused together (Additional file 2: Figure S2b). Landmark structures that contain some of the identified molecular components were marked by different colors on cytological maps (Figures 2b, e). As can be seen from example of the *P. ridibundus* LBC H, the immunofluorescence assay with antibodies K121 against TMG-cap of small nuclear RNA revealed marker loops accumulating spliceosomal components (Figure 2a). One can easily identify these loops on lampbrush chromosome spreads by brilliant staining. Immunostaining with antibodies against nucleolus protein Nopp-140 showed presence of one nucleolus of approximately 10 μm in diameter in association with the nucleolus organizing region (NOR) on LBC H (Figure 2c). Using fluorescence *in situ* hybridization (FISH) we located pericentromeric satellite RrS1 repeat, which was first identified by Ragghianti and coauthors [27], in *P. ridibundus* and *P. lessonae* from Poland. Furthermore, FISH with oligonucleotide probe specific to (TTAGGG)_n_ repeat in its turn allowed to mark interstitial blocks of this repeat or sequences containing TTAGGG repeat on constructed map of LBC H (Figure 2d). Using antibodies against coilin we distinguished and marked on the LBC H map coilin-positive granules, which were found not only in telomere regions, but also in interstitial sites corresponding to chromomeres containing (TTAGGG)_n_ repeat (Figure 2g). Cytological maps of other lampbrush chromosomes were constructed in the same way.

The results of detailed characterization of lampbrush chromosomes of *P. ridibundus* and *P. lessonae* as well as comparative analysis of the orthologs with special emphasize on difference between sets of marker structures are presented below.

### *Comparison of* P. ridibundus *and* P. lessonae *lampbrush chromosome A*

**Figure 3:**
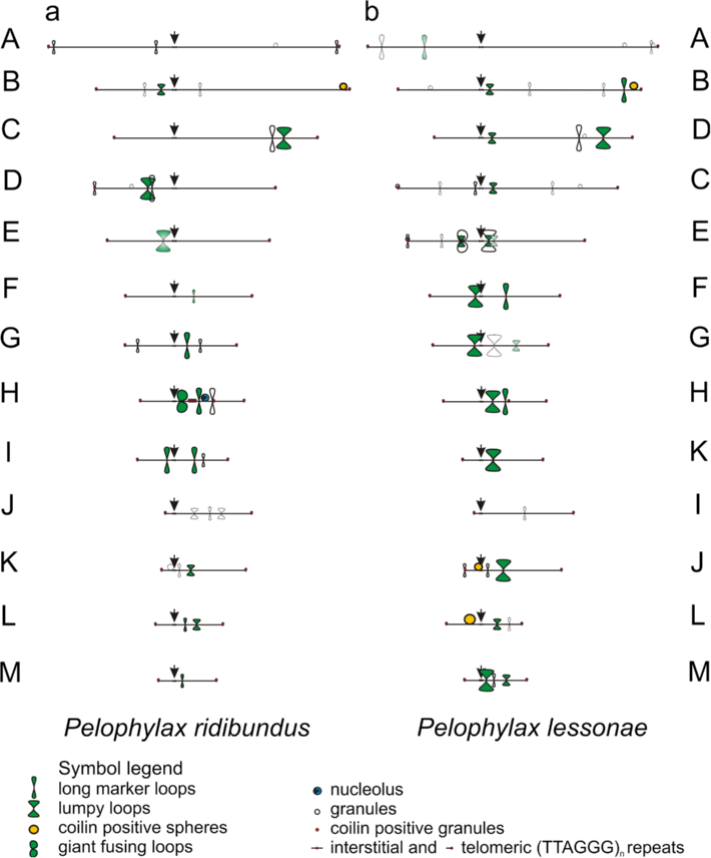
**Cytological maps of lampbrush chromosomes of both parental species of *Pelophylax esculentus *complex.** Working maps of all lampbrush chromosomes of *P. ridibundus* (**a**) and *P. lessonae* (**b**). Chromosomes were arranged and lettered according to their relative length. Comparative locations of the most conspicuous landmark structures colored according to their marker components are shown. Green color indicates accumulation of pre-mRNA splicing factors, red – coilin positive structures, yellow – accumulation of both splicing factors and coilin, blue – enrichment with nucleolus components.

In karyotypes of both species, chromosome A is the longest chromosome at the lampbrush stage (Tables 1 and 2). It is characterized by presence of several landmark structures namely lateral loops with special morphology and composition. In subtelomeric regions of the long and the short arms of LBC A of both species, we identified noticeable marker loops that do not differ from simple lateral loops in terms of concentration of snRNPs (Figures 1, 3 and Additional file 1: Figure S1). These loops extend to 10 μm in length being 1.5 times longer than the vast majority of simple lateral loops. The lampbrush chromosome A of *P. lessonae* bears a marker loop in its short arm, which accumulates splicing factors (Figure 3b). This loop was not recognizable in the orthologous lampbrush chromosome of *P. ridibundus*. Instead, the chromosome A of the latter bears another pair of marker loops on the short arm not far from centromeric region (Figures 1, 3a and Additional file 1: Figure S1). This particular marker loop does not accumulate splicing factors but is characterized by unusual morphology of RNP-matrix. On the majority of preparations, distinctive chromomeres were found in the long arm of LBC A (Figures 1, 3a, b and Additional file 1: Figure S1). These chromomeres occupy slightly different positions in corresponding lampbrush chromosomes of both species. In addition, chromosome A of *P. ridibundus* sometimes has a large chromomere in subtelomeric region. In our opinion, distinctive chromomeres are not reliable for identification of lampbrush chromosome A, since they are not always present in their usual positions.

### *Comparison of* P. ridibundus *and* P. lessonae *lampbrush chromosome B*

**Figure 4:**
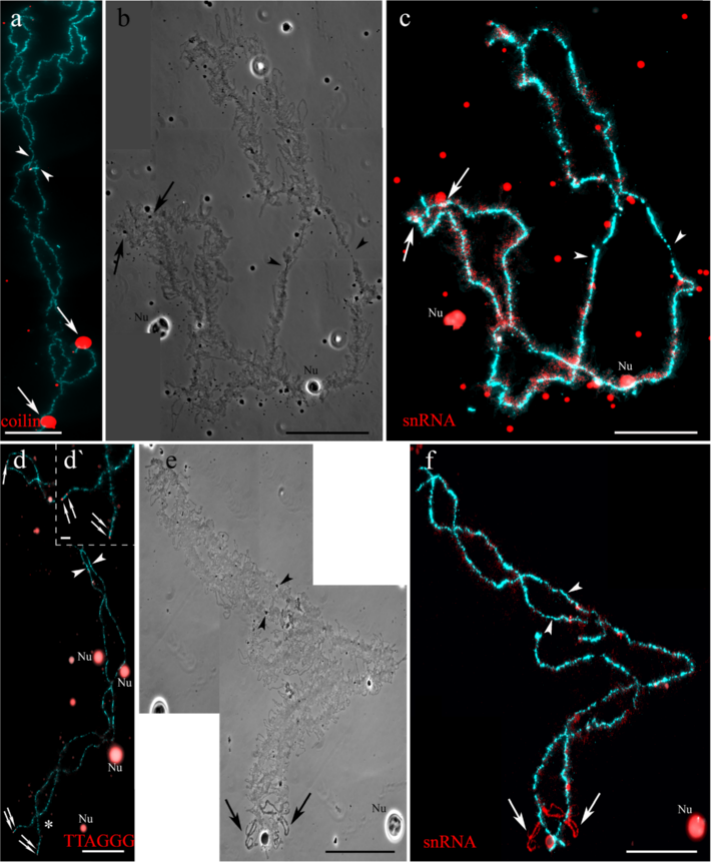
**Comparison of *P. ridibundus *(a, b, c, d, d`) and *P. lessonae *(e, f) lampbrush chromosome B.** Phase contrast micrographs (**b, e**), immunofluorescent staining with antibodies against coilin (**a**), TMG-cap of snRNAs (**c, f**). Sites with mapped (TTAGGG)_n_ repeat (**d**) are indicated by arrows. Spheres (shown by arrows) in subtelomeric region of the long arm of *P. ridibundus* lampbrush chromosome B (**a, b, c**). Long marker loops (shown by arrows) (**e, f**) close to sphere loci in the long arm of *P. lessonae* lampbrush chromosome B. Chromosomes are counterstained with DAPI. Nu – extrachromosomal nucleoli. Arrowheads show centromeres. Scale bars = 50 μm. Asterisks indicate enlarged fragment of lampbrush chromosome B with mapped (TTAGGG)_n_ repeat (**d**`). Arrows indicates telomeric and interstitial blocks of sequences containing (TTAGGG)_n_ repeat in a subtelomeric region of the long arm of *P. ridibundus* lampbrush chromosome B (**d**'). Scale bars = 10 μm.

In both species, the long arm of chromosome B has a sphere in its subtelomeric region, which contains pre-mRNA splicing factors and protein coilin (Figures 4a, b, c, e, f). There is an interstitial block of (TTAGGG)_n_ repeat or sequences containing (TTAGGG)_n_ repeat near this sphere in *P. ridibundus* but not *P. lessonae* lampbrush chromosome B (Figures 4d, d`, 3a, b). Another distinctive feature of *P. lessonae* LBC B is a pair of long marker loops containing pre-mRNA splicing factors in its long arm (Figures 4e, f, 3b). Notably, there are no loops in the same locus of the *P. ridibundus* lampbrush chromosome B. We have also registered appearance of distinctive chromomere in the short arm of *P. lessonae* LBC B on the majority of preparations.

### *Comparison of* P. ridibundus *lampbrush chromosome С and* P. lessonae *lampbrush chromosome D*

Lampbrush chromosome С of *P. ridibundus* and chromosome D of *P. lessonae* differ in their comparative length, but have almost identical centromeric index and similar pattern of marker structures (Additional file 3: Figures S3a, b, c, d). Both lampbrush chromosomes contain marker loop, which does not accumulate splicing factors, and a conspicuous lumpy loop enriched with spliceosomal snRNPs (Figures 3a, b). Distance between these loops is slightly different being longer in *P. lessonae*. On some preparations, a large chromomere can localize between mentioned loops in LBC D of *P. lessonae*. In addition, a smaller lumpy loop accumulating splicing factors is situated near centromeric region in the long arm of *P. lessonae* lampbrush chromosome D but not in *P. ridibundus* lampbrush chromosome C (Additional file 3: Figures S3a, b, c, d).

### *Comparison of* P. ridibundus *lampbrush chromosome D and* P. lessonae *lampbrush chromosome C*

Lampbrush chromosome D of *P. ridibundus* is similar to chromosome C of *P. lessonae* according to its centromeric index (Tables 1 and 2). Nevertheless they have a lot of dissimilarities in overall structure, type of marker loops and their arrangement (Additional file 3: Figures S3e, f, g, h). In the short arm of lampbrush chromosome D of *P. ridibundus*, there are lumpy loops accumulating snRNPs and long marker loops that do not concentrate them. Another set of long marker loops without enrichment in splicing factors was also found in terminal region of the short arm of chromosome D in this species (Additional file 3: Figures S3e, f). Large chromomere is located in the short arm of *P. ridibundus* chromosome D on the majority of lampbrush chromosome preparations. Lampbrush chromosome C of *P. lessonae* contains a terminal granule and marker loops that do not accumulate spliceosomal components in the short arm near the centromere. In addition, lumpy loops containing splicing factors form not far from centromere in the long arm of LBC C of *P. lessonae* (Additional file 3: Figures S3g, h).

### *Comparison of* P. ridibundus *and* P. lessonae *lampbrush chromosome E*

There are a few marker structures on lampbrush chromosome E of *P. ridibundus*: only in several lampbrush chromosome spreads, we observed lumpy loops accumulating splicing factors in the short arm (Table 1 and Figure 3a). Similarly, lampbrush chromosome E of *P. lessonae* has a pair of lumpy loops with splicing factors in its short arm, although there are a prominent granule and a pair of marker loops in the terminal region of the short arm (Table 2 and Figure 3b). We also identified lumpy loops containing pre-mRNA splicing factors that appear in the long arm near the centromere in the *P. lessonae* lampbrush chromosome E (Figure 3b). All lumpy loops reached giant size in three lampbrush chromosome spreads of the *P. lessonae* oocytes.

### *Comparison of* P. ridibundus *and* P. lessonae *lampbrush chromosome F*

**Figure 5:**
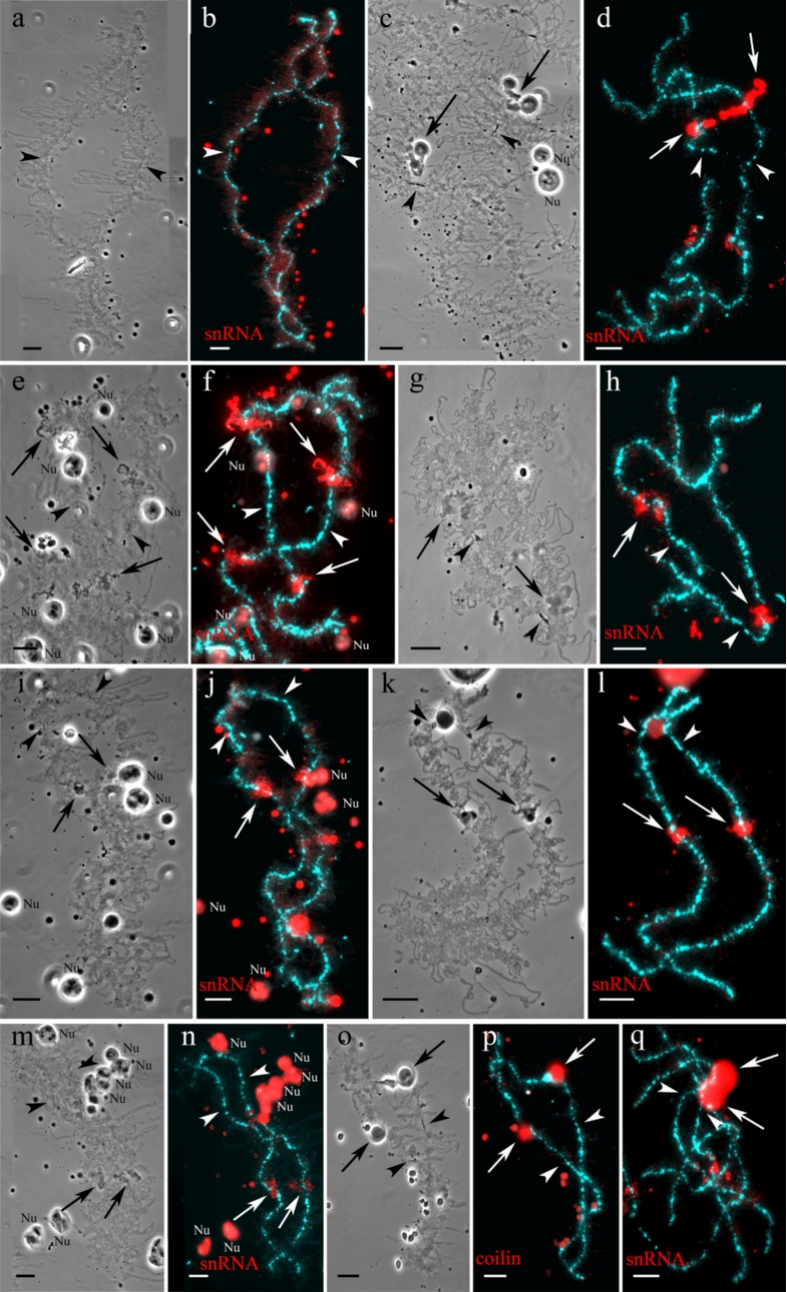
**Comparison of *P. ridibundus *lampbrush chromosomes F (a, b), I (e, f), K (i, j), L (m, n) and *P. lessonae *lampbrush chromosomes F (c, d), K (g, h), J (k, l) and L (o, p, q).** Phase contrast micrographs (**a, c, e, g, i, k, m, o**) and immunofluorescent staining with antibodies against snRNA (**b, d, f, h, j, l, n, q**) and coilin (**p**). Chromosomes are counterstained with DAPI. Arrows indicate the most conspicuous marker structures on lampbrush chromosomes, arrowheads show centromeres. Nu – extrachromosomal nucleoli. Scale bars = 10 μm.

Similar to chromosome E, lampbrush chromosome F of *P. ridibundus* is typically devoid of any marker structures (Figures 5a, b). Rarely, a marker loop with splicing factors can appear in the long arm of this chromosome. Lampbrush chromosome F of *P. lessonae* usually bears similar but more prominent marker loops at the same region (Figures 5c, d). Presence of a giant lumpy loop accumulating splicing factors is typical for the short arm of *P. lessonae* LBC F but not for the short arm of orthologous chromosome of *P. ridibundus*.

### *Comparison of* P. ridibundus *and* P. lessonae *lampbrush chromosome G*

Lampbrush chromosome G of *P. ridibundus* has a pair of morphologically distinct marker loops in the short arm. Giant marker loops accumulating splicing factors are situated in the long arm of this chromosome, and marker loops with normal amount of splicing factors locate near the telomeric region (Additional file 3: Figures S3i, j). As opposed to *P. ridibundus* LBC G, the ortologous lampbrush chromosome of *P. lessonae* is characterized by giant lumpy loops in the short arm near the centromere (Additional file 3: Figures S3k, l). In some preparations, we observed giant lumpy loops situated near the centromere in the long arm of chromosome G. Another pair of lumpy loops can be located close to chromosomal terminal region. All lumpy loops of *P. lessonae* LBC G accumulate pre-mRNA splicing factors.

### *Comparison of* P. ridibundus *and* P. lessonae *lampbrush chromosome H*

Differences in the morphology of LBC H between the two parental species are more essential. Lampbrush chromosome H of *P. ridibundus* bears a pair of giant fusing loops, which accumulate splicing factors, on the long arm (Figures 2a, b, f and Additional file 2: Figures S2a, b). Apart from these loops, there are two sets of marker loops in the long arm and an obvious chromosome-associated nucleolus (Figures 2b, c and Additional file 2: Figures S2a, b). The nucleolus has been identified by immunofluorescent staining with antibodies against its canonical components – proteins Nopp-140, No38 and fibrillarin (Figures 2b, c, e). One type of the marker loops is enriched with splicing factors, while the other one is not. In addition, we have detected two interstitial blocks of (TTAGGG)_n_ repeat or another longer repeat containing TTAGGG motif (Figures 2d, e). The first block is especially long and locates between giant fusing loops and marker loop accumulating spliceosome components, and the second one is relatively small and is located near the second group of landmarks. The orthologous lampbrush chromosome in *P. lessonae* has a somewhat different morphology (Additional file 4: Figures S4a, b). Lumpy loops and marker loops with splicing factors are located in the long arm of this chromosome, but chromosomal nucleolus organizer region remains inactive and nucleolus does not develop. In contrast with *P. ridibundus* chromosome H, in the LBC H of *P. lessonae*, interstitial telomeric (TTAGGG)_n_ repeat or sequences containing TTAGGG repeat are present as a single block, which is located near the marker loop in the long arm (Additional file 4: Figures S4c, d).

### *Comparison of* P. ridibundus *lampbrush chromosome I and* P. lessonae *lampbrush chromosome K*

Lampbrush chromosome I of *P. ridibundus* and chromosome К of *P. lessonae* differ from each other not only by comparative length, but also in the arrangement of marker structures (Figures 5e, f, g, h, 3a, b). According to specific pattern of immunostaining with mAbs K121 and Y12, *P. ridibundus* long and short arms of LBC I bear two pairs of marker loops accumulating splicing factors. In addition to these unusual loops, there is a pair of marker loops that do not accumulate splicing factors near the terminal region of the long arm (Figures 5e, f, 3a). In its turn, *P. lessonae* LBC K has a single pair of marker loops with higher concentration of splicing factors in its long arm. At the same time there are no long marker loops in the short or long arms of *P. lessonae* LBC K (Figures 5g, h, 3b).

### *Comparison of* P. ridibundus *lampbrush chromosome J and* P. lessonae *lampbrush chromosome I*

Lampbrush chromosome J of *P. ridibundus* and chromosome I of *P. lessonae* have almost identical centromeric index despite difference in the relative length (Tables 1 and 2) (Figure 3). Marker structures on this lampbrush chromosome in both species are weakly visible and can be absent in some oocytes. There is no much difference between the two orthologous lampbrush chromosomes in the parental species. Lampbrush chromosome J of *P. ridibundus* occasionally contains two pairs of small lumpy loops with a pair of long marker loop between them. All loops mapped do not accumulate splicing factors if compared with normal lateral loops (Figure 3a). Sometimes, lampbrush chromosome I of *P. lessonae* bears a pair of marker loops in a similar position, that do not accumulate pre-mRNA splicing factors (Figure 3b).

### *Comparison of* P. ridibundus *lampbrush chromosome K and* P. lessonae *lampbrush chromosome J*

Lampbrush chromosomes K of *P. ridibundus* and J of *P. lessonae* are also characterized by similar centromeric index (Figures 5i, j, k, l). *P. ridibundus* LBC K bears a sphere close to centromere in the short arm and marker loops in the long arm. We revealed a pair of lumpy loops accumulating splicing factors in the long arm of this chromosome in all lampbrush chromosome spreads (Figures 5i, j). In the same locus of *P. lessonae* LBC J, we always identified lumpy loops accumulating snRNPs, marker loops with unusual morphology and a sphere containing pre-mRNA splicing factors and protein coilin. In addition, a pair of marker loops without higher concentration of spliceosomal components was detected in the terminal region of the short arm of *P. lessonae* LBC J (Figures 5k, l).

### *Comparison of* P. ridibundus *and* P. lessonae *lampbrush chromosome L*

The long arm of lampbrush chromosome L of *P. ridibundus* is characterized by lumpy and marker loops enriched with splicing factors (Figures 5m, n). Similar lampbrush chromosome in *P. lessonae* contains lumpy loops that do not attract higher amounts of splicing factors (Figures 5o, q). In the short arm of *P. lessonae* LBC L, we found a large sphere containing coilin and snRNAs typical for a group of Cajal body – like bodies (Figures 5o, p).

### *Comparison of* P. ridibundus *and* P. lessonae *lampbrush chromosome M*

Chromosome M in *P. ridibundus* and its ortholog in *P. lessonae* represent the smallest chromosomes at the lampbrush stage (Figure 1 and Tables 1, 2). The established pattern of distribution of splicing factors demonstrated that these chromosomes also have some distinctive features in marker structures arrangement. A pair of marker loops accumulating splicing factors forms in the long arm of *P. ridibundus* LBC M. At the same time *P. lessonae* LBC M bears large and small lumpy loops with higher concentration of splicing factors, and also long marker loops between them, which do not accumulate splicing factors in their RNP-matrix (Figure 3).

All data presented are summarized on the constructed cytological maps of all *P. ridibundus* and *P. lessonae* lampbrush chromosomes (Figures 3).

### High-resolution mapping and analysis of transcriptional activity of telomere and centromere repeats

**Figure 6:**
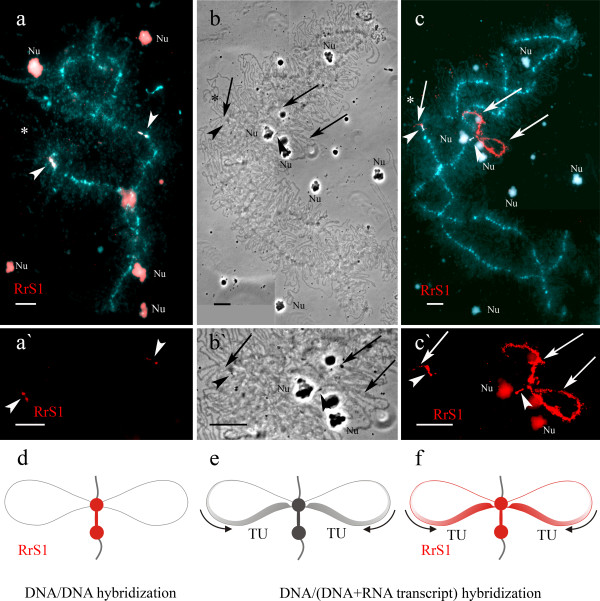
**Localization and transcriptional activity analysis of RrS1 repeat in centromere regions of *P. lessonae *lampbrush chromosomes. a**, **c**. Microphotographs with DNA/DNA (**a**) and DNA/(DNA+RNA) (**c**) FISH of RrS1 repeat to lampbrush chromosomes of *P. lessonae* (red). Chromosomes are counterstained with DAPI (blue). Asterisks indicate enlarged fragments of the same chromosomes with fluorescent signal of RrS1 repeat shown on panels **a`** and **c`**. **d, f**. Schematic drawings of chromosomal centromere regions demonstrate the distribution of visualized DNA/DNA (**d**) and DNA/(DNA+RNA) (**f**) FISH signals (red). Transcripts of RrS1 repeat are not detectable after pre-treatment with RNase A; RrS1 repeat localizes in two distinctive chromomeres in a centromere region (indicated by arrowheads) (**a, a`, d**). Without pre-treatment with RNase A hybridization signal is clearly revealed not only in centromere chromomeres but also in RNP-matrix of long lateral loops (indicated by arrows) extended from the centromere chromomeres (**c, c`, f**). Circular arrows show direction of transcription (**e, f**). TU – transcriptional unit. **b, b`, e**. Corresponding phase contrast micrographs (**b, b`**) and schematic drawing of a centromere region (**e**). Nu – extrachromosomal nucleoli. Scale bars = 10 μm.

Fluorescent *in situ* hybridization (FISH) was applied to determine the localization of centromeric RrS1 tandem repeat in chromosomes of water frog species from the Eastern Ukraine. In both metaphase and lampbrush *P. ridibundus* chromosome preparations, RrS1 probe hybridized to all 13 chromosomes (Additional file 5: Figure S5a), however, chromosomes varied in the fluorescence signal intensity. Similarly, *P. lessonae* metaphase chromosomes showed almost the same pattern of RrS1 repeat distribution, but fluorescence signal was not detectable in one small chromosome (Additional file 5: Figure S5b). In *P. lessonae* lampbrush chromosome spreads, all chromosomes had signal of varying intensity (Additional file 6: Figure S6). In lampbrush chromosome preparations of the both species, the signal from hybridized probe was located within the centromere regions at two distinctive chromomeres as well as a constriction between them (Figures 6a, a`).

Taking into account presence of lateral loops with long transcription units in centromere regions of lampbrush chromosomes of the lake frog and the pool frog, we checked for transcriptional activity of RrS1 repeat. Using DNA/(DNA+RNA) FISH we have detected transcripts of RrS1 in the centromere regions of the majority of lampbrush chromosomes in both species (Figures 6b, b`, c, c`, e, f). In our control experiments, RNAse treatment eliminated FISH signals on the lateral loops. To confirm the specific hybridization of the labeled probe to the nascent transcripts we performed FISH according to DNA/RNA hybridization protocol, in which chromosomal DNA was not denatured and RNAse treatment was also omitted. After DNA/RNA FISH we observed bright fluorescence signal only in the RNP matrix of lateral loops, emerging from large centromeric chromomeres. It is important to note that when chromosomes are not denatured FISH with RrS1 repeat specifically reveals RNA molecules within transcription units on specific lateral loops of frog lampbrush chromosomes and does not label other chromosomal segments (chromomeres and other loops). Observation of DNA/RNA hybrids on RNP-matrix of lateral loops of lampbrush chromosomes during FISH experiments serves as a clear cytological evidence of satellite DNA transcription [28].

**Figure 7:**
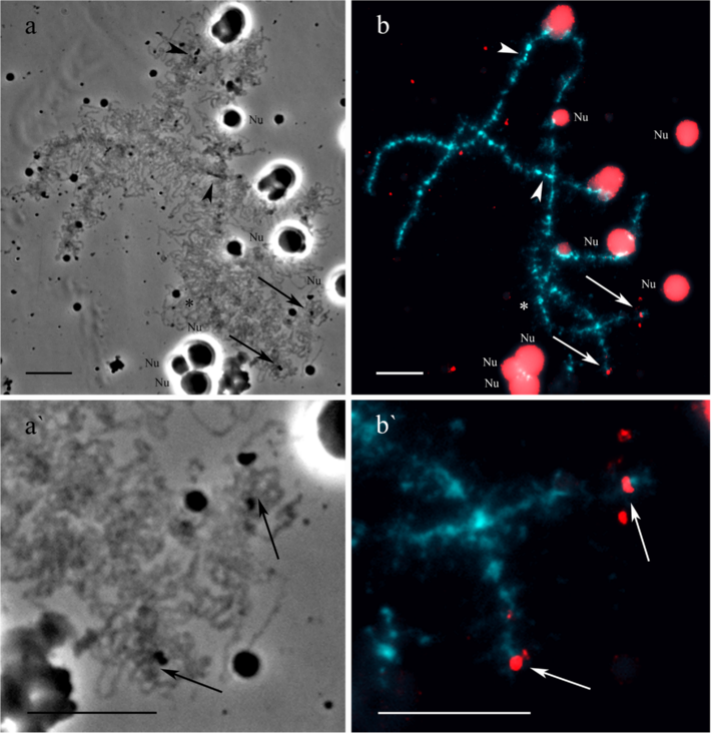
**Detection of telomere repeat transcripts at the terminal regions of *P. ridibundus *lampbrush chromosomes.** DNA/RNA FISH with (TAACCC)_5_-biotin on *P. ridibundus* lampbrush chromosome E (**b, b`**). (TTAGGG)_n_-repeat transcripts are visible in small caps at the ends of lampbrush chromosomes (indicated by arrows). Asterisk indicates enlarged region of lampbrush chromosome E. Chromosomes are counterstained with DAPI. Corresponding phase contrast micrographs are shown (**a, a`**). Arrowheads indicate centromeres. Nu – extrachromosomal nucleoli. Scale bars = 10 μm.

To determine the localization of TTAGGG repeats we performed DNA/DNA-hybridization with (TTAGGG)_5_ oligonucleotide. Results demonstrated that TTAGGG repeat is situated in the terminal chromomeres of all lampbrush chromosomes and in interstitial sites of chromosomes B and H (Figures 2d, 4d, d` and Additional file 4: Figure S4d). In birds, telomeric repeat is transcribed during the lampbrush chromosome stage of oogenesis [29,30], but there is still lack of data on its activity in amphibian lampbrush chromosomes. Therefore, we have analyzed the transcriptional activity of telomeric repeat on lampbrush chromosomes of *P. ridibundus* and *P. lessonae*. DNA/RNA FISH with single stranded oligonucleotide probes to C- and G-rich strands of telomeric TTAGGG repeat allowed to discover telomere repeat transcripts on lampbrush chromosomes of both *P. ridibundus* and *P. lessonae* (Figures 7a, a`, b, b`). The phenomenon was observed in all analyzed lampbrush chromosome sets from various individuals. TTAGGG repeat transcripts were localized in small caps at the ends of lampbrush chromosomes, and as opposed to RrS1 repeat transcripts, they did not form long transcriptional units. The average size of chromosomal caps enriched with transcripts of TTAGGG repeat was about 1.5 μm. Notably, these telomeric repeat containing transcripts can be detected only by C-rich single stranded oligonucleotide probe (TAACCC)_5_ (Figures 7b, b`) but not by the G-rich (TTAGGG)_5_ oligonucleotide.

## Discussion

### Distinctive features of lampbrush chromosomes of *P. ridibundus* and *P. lessonae* from Eastern Ukraine

Analysis of full sets of giant lampbrush chromosomes from oocytes of two parental species of the *P. esculentus* complex allows to study mechanisms underlying the maintenance of water frog population systems [16,25]. Here we characterized in detail various marker structures on lampbrush chromosomes of *P. ridibundus* and *P. lessonae* water frogs from Eastern Ukraine, localized nucleolus organizer region, coilin-enriched bodies, centromeres and interstitial sites of telomere repeat. These results were summarized in cytological maps for the first time constructed for all 13 lampbrush chromosomes of the both species.

Constructed maps clearly demonstrated dissimilarities in number and distribution of marker structures of lampbrush chromosomes between parental species. We conclude lampbrush chromosomes of *P. ridibundus* and *P. lessonae* from Eastern Ukraine to have four main differences. Firstly, orthologous *P. ridibundus* and *P. lessonae* lampbrush chromosomes differ in positions of centromeres and comparative length, the latter could be the consequence of various levels of chromatin decondensation. Secondly, *P. ridibundus* and *P. lessonae* lampbrush chromosomes differ in number and localization of marker structures such as long marker and lumpy loops. In our opinion, the most reliable way to identify individual parental lampbrush chromosomes in karyotypes of hybrid frogs would be detection of the special loops accumulating splicing factors, because the pattern of these marker loops differs across all orthologous chromosomes. Thirdly, *P. ridibundus* and *P. lessonae* lampbrush chromosomes differ in terms of nucleolus formation. *P. ridibundus* LBC H bears associated nucleoli, but none of *P. lessonae* lampbrush chromosomes has active chromosomal NOR. Fourthly, *P. ridibundus* and *P. lessonae* chromosomes differ by the presence of interstitial blocks of the sequences containing (TTAGGG)_n_ fragment. Presence of the (TTAGGG)_n_ sequence in interstitial sites allows to distinguish chromosomes B and H of *P. ridibundus* from orthologous chromosomes of *P. lessonae*.

Interpopulation similarities in the morphology of certain lampbrush chromosomes were also noticed. We compared the morphology of lampbrush chromosomes of *P. ridibundus* and *P. lessonae* from Kharkov region with the morphology of lampbrush chromosomes of these species from Poland (Table 3), the latter being described earlier by Bucci et al. [16]. We found, for instance, that localization of spheres is similar in LBCs 2 and 9 of *P. ridibundus* and *P. lessonae* from Poland and homologous lampbrush chromosomes of the corresponding species from Kharkov region. In addition, homology was seen in LBC 10 of *P. ridibundus* from Poland population and LBC H of *P. ridibundus* from Kharkov region (Table 3). Both chromosomes contain active NOR and a number of giant loops in similar loci. Moreover, localization of all marker structures in lampbrush chromosomes 2, 9 and 10 of *P. ridibundus* from Poland is analogous to localization of marker structures on corresponding lampbrush chromosomes of *P. ridibundus* from Greece and some other species of the genus *Pelophylax*[25,31].

**Table 3:** **Comparison of lampbrush chromosomes of *P. ridibundus *from Poland and Kharkov region**

**Lampbrush chromosomes of *P. ridibundus *from Poland**		**Lampbrush chromosomes of *P. ridibundus *from Kharkov region**	
**Number of LBC according to relative length**	**Marker structures**	**Number of LBC according to relative length**	**Marker structures**
I	long arm: s/t granule	A	short arm: i/c long marker loop, s/t long marker loop; long arm: s/t long marker loop
II	long arm: i/c bush like loop, s/t sphere	B	short arm: i/c lumpy loop; long arm: s/t sphere
III		D	short arm: t long marker loop, i/c lumpy loop, i/c long marker loop
IV	long arm: s/t dense like loop	C	long arm: s/t long marker loop, i/c lumpy loop
V		E	short arm: rarely i/c lumpy loop
VI		F	long arm: rarely i/c long marker loop
VII	long arm: i/c giant sructures	G	short arm: i/c long marker loop; long arm: usually i/c 1 sometimes i/c 2 long marker loops,
VIII*	long arm: i/c bush like	J*	
IX	short arm: sphere; long arm: i/c dense like	K	short arm: sometimes sphere; long arm: usually i/c 1 sometimes i/c 2 lumpy loops,
X	long arm: i/c giant structures, no	H	long arm: i/c giant fusing loop, i/c 2 long marker loops, no
XI*		I*	short arm: i/c long marker loop; long arm: i/c 2 marker loops
XII*		L*	long arm: i/c lumpy loop, i/c long marker loop
XIII*		M*	long arm: i/c long marker loop

* - The correlation between the particular lampbrush chromosomes is established only according to their relative length.

Abbreviations:

s/t - subtelomeric position of marker structure.

t - telomeric position of marker structure.

i/c - intercalary position of marker structure.

We also observed intraspecific differences in localization of marker structures in lampbrush chromosomes of the same species from various populations. For instance, LBC 10 of *P. lessonae* from Poland population bears one attached nucleolus [16], but neither homologous LBC H nor any other lampbrush chromosome of *P. lessonae* from Kharkov region contains active chromosomal NOR. Additionally, we also determined dissimilarities in positions of long marker and lumpy loops. In contrast with lampbrush chromosomes of *P. lessonae* from Kharkov region, lampbrush chromosomes of *P. lessonae* from Poland were characterized by a number of giant fusing loops. Dissimilarities in localization of complex loops on lampbrush chromosomes are the consequences of intraspecies variation and polymorphism of underlying genomic sequences.

### Marker structures on lampbrush chromosomes of *P. ridibundus* and *P. lessonae*

Molecular composition of marker structures on lampbrush chromosomes of *P. ridibundus* and *P. lessonae* from Kharkov region provides information on their nature. Particularly, the long marker loops developing in terminal and interstitial regions of almost all lampbrush chromosomes in both species could be classified into two types: some of them accumulate pre-mRNA splicing factors, while others do not. The transcripts synthesized on these marker loops with dense RNP-matrix are unknown since the genomes of the lake frog and the pool frog are still poorly investigated. At the same time, it can be assumed that RNA content of complex loops is most probably represented by non-coding transcripts and even transcripts of tandem repeats (reviewed in [22]). It is known for avian lampbrush chromosomes that transcripts of satellite DNA participate in formation of loops enriched with splicing factors. Particularly, DNA sequences responsible for lumpy loop formation in the long arm of chicken LBC 2 are shown to be represented by tandem “lumpy loop” 2 repeat (LL2R) [32]. Intriguingly, lumpy loops in this locus attract high amounts of splicing factors, which could be explained by presence of potential binding sites for the spliceosome components in the LL2R transcript. Another example of prominent marker structures, which we observed on lampbrush chromosomes of water frogs from the Seversky Donets River basin, are giant fusing loops that form on LBC H of *P. ridibundus*. One of the possible explanations implies the nascent RNA transcripts on giant fusing loops might also derive from highly repeated non-coding DNA sequences.

As far as the chromosome associated nuclear bodies are concerned, one of the most prominent examples is spherical nucleolus that forms on the long arm of LBC H of the *P. ridibundus*. Neither orthologous chromosome nor any other lampbrush chromosomes of *P. lessonae* from Kharkov region carry attached nucleolus meaning that chromosomal NOR is completely inert at this stage of oogenesis. We have not observed any difference in NOR activity on LBC H throughout all seasons of the year. At the same time, generally the activity of ribosomal genes in oocyte nuclei of water frogs and other amphibians is not inhibited, since a great number of extrachromosomal nucleoli are always present in germinal vesicles of these species ([23]; our observations). Similar phenomenon of differential inactivation of main clusters of ribosomal genes on lampbrush chromosomes was described in oocytes of crested newts [28]. This selective inhibition of chromosomal NORs, but not the amplified ones that are genetically identical, can be mediated by short interfering RNA – depending and long non-coding RNA – depending mechanisms that are known to be involved into nucleolar dominance in plants and rRNA gene silencing in mammals respectively [33,34].

Another type of lampbrush chromosome associated bodies is the histone locus body that is characterized by presence of coilin [35]. It was established in *Xenopus* that histone locus bodies form in association with clustered histone genes upon their activation [36,37]. In addition to coilin, histone locus bodies also accumulate U7 snRNA, symplekin, FLASH and other components involved into 3^′^-processing of histone pre-mRNA. Coilin- and snRNA-rich spherical structures were found in spread content of *P. ridibundus* and *P. lessonae* oocyte nuclei and were attached to specific loci on lampbrush chromosomes. Terminal granules were detected at the ends of all lampbrush chromosomes of both parental species and were shown to accumulate coilin. We demonstrated colocalization of coilin-positive granules with either terminal or interstitial sites of DNA sequences containing TTAGGG motif. In human somatic cells, coilin-positive bodies play an important role in telomerase maturation, storage and its delivery to telomeres [38,39]. We speculate that coilin-positive granules at the telomeres of lampbrush chromosomes can be involved into telomerase dynamics.

Interstitial blocks of TTAGGG repeat or sequences containing this repeat were detected in LBCs B and H of *P. ridibundus* and chromosome H of *P. lessonae*. Interstitial blocks of TTAGGG sequences can arise as a result of chromosomal rearrangements or telomere-telomere fusion (reviewed by [40]). TTAGGG repeat can be also included in other tandemly repetitive sequences as it was found in cases of GS repeat of chaffinch and cen1 repeat of chicken [41,42]. They probably could originate via reparation of double-stranded breaks. In the subtelomeric region of *P. ridibundus* chromosome B, interstitial site of TTAGGG repeat could appear as a result of unequal recombination or gene conversion event as shown for some chromosomes in human karyotype [40]. Since *P. ridibundus* and *P. lessonae* lampbrush chromosome H have similar pattern of small (TTAGGG)_5_ hybridization signal, we suppose that interstitial site of TTAGGG repeat appeared in this locus in their common ancestor. The other longer interstitial block of TTAGGG sequence in LBC H was found only in *P. ridibundus* karyotype, which suggests that it appeared after *P. ridibundus* and *P. lessonae* divergence.

In lampbrush chromosomes of *P. ridibundus* and *P. lessonae* from Kharkov region, centromeres are well identified as dense enlarged chromomeres. In contrast, in lampbrush chromosomes of *P. lessonae* from Poland, centromeres were not visible on phase contrast microphotographs [16]. The evident difference in the morphology of lampbrush centromeres supposedly depends on the amount of centromeric satellite repeats such as RrS1 repeat [27]. The FISH approach confirmed that all centromeres of *P. lessonae* from Kharkov region have large clusters of centromeric RrS1 repeat if compared to centromeres of *P. lessonae* from Poland.

### Transcription of telomere and centromere repeats on lampbrush chromosomes of *P. ridibundus* and *P. lessonae*

As it has been shown in a number of studies, termini of amphibian lampbrush chromosomes do not carry any conspicuous loops which can be analogous to terminal loops typical for avian lampbrush chromosomes (reviewed in [22,43]). In fact, in the very terminal transcription units of lampbrush chromosomes of chicken, turkey and pigeon, G-rich transcripts of (TTAGGG)_n_ repeat were revealed by FISH that was the first demonstration of telomere repeat transcription in vertebrates [29,30,44]. Here we provide, to our knowledge for the first time, evidences in favor of telomere repeat transcription during the lampbrush stage of oogenesis in amphibian species. G-rich telomeric repeat transcripts were detected on lampbrush chromosomes of water frogs as small transcription units or even tiny little caps. Using FISH of strand-specific oligonucleotides to nascent RNA transcripts we have also determined the direction of (TTAGGG)_n_-repeat transcription that in water frog lampbrush chromosomes proceeds to the chromosomal end. Resulting nascent transcripts of telomere repeat stay associated with terminal chromomeres for a long time.

General properties of telomere repeat transcripts on lampbrush chromosomes of water frogs and birds are the same: both amphibian and avian (TTAGGG)_n_-repeats are transcribed only from C-rich strand in a direction towards the end of a chromosome [29, 30; our data], which could point on similarities of their functions.

Non-coding telomere repeat transcripts named TERRA (telomeric repeat-containing RNA) with the same characteristics were later described in human and mouse somatic cells and in budding yeast (reviewed by Luke and Lingner, [45]). In mammals, TERRA transcripts originating from regulated promoters consist of UUAGGG repeated sequence and a subtelomeric sequence [46]. TERRA are proposed to take part in heterochromatinization of telomere regions by RNAi mechanism and to form quadruplex structures that stabilize telomere. TERRA also might regulate replication of telomeres and inhibit telomerase activity [45]. Significance of active transcription of telomere DNA, which takes place during the lampbrush chromosomes stage of female meiosis, is largely unknown. However, we suppose that highly decondensed lampbrush chromosomes is a convenient model for studying the phenomenon of telomere repeat transcription owing to fine cytological resolution and opportunity to visualize active terminal transcription units and their RNP-content.

Intensive transcription of satellite repeats that leads to formation of extended lateral loops emerging from chromomeres is also a distinctive feature of the lampbrush stage of oogenesis [47,48]. Transcripts of pericentromere satellite DNA were discovered in both amphibian and avian lampbrush chromosomes several decades ago long before the breakthrough in the mechanisms of genome silencing via regulatory non-coding RNA [48-54]. Subsequently, transcripts of pericentromeric and centromeric tandem repeats were also revealed in actively proliferating somatic cells of various animals including mammals [55].

We demonstrated the transcription of satellite RrS1 repeat in the centromere regions of lampbrush chromosomes of *P. lessonae* and *P. ridibundus* using classical and accredited approach (Figures 6c, c`, f). To our knowledge, our results represent the first example of transcription of clustered centromeric satellite DNA in lampbrush chromosomes of Anura.

Phenomenon of satellite DNA transcription in lampbrush chromosomes of water frogs can be hypothetically explained by a regulatory role of maternal non-coding RNA in early stages of embryogenesis (reviewed in [43,48]). It is tempting to speculate that transcripts of telomeric, subtelomeric, pericentromeric and interstitial arrays of tandemly repetitive sequences synthesized during the lampbrush stage of oogenesis can be used as regulatory RNA molecules after fertilization. Such molecules could be employed for RNA-depending inhibition of transcription in definite chromosomal loci and heterochromatinization during early stage of embryogenesis providing additional mechanism for genomic stability and maintaining the integrity of species.

## Conclusions

In order to characterize the lampbrush karyotypes of parental species of the *P. esculentus* complex, we have constructed detailed working maps of all lampbrush chromosomes of *P. ridibundus* and *P. lessonae* originating from Eastern Ukraine. These maps contain information about comparative location of the most conspicuous landmark structures classified according to their marker components and about the positions of centromere and interstitial (TTAGGG)_n_-repeat sites. Furthermore, we demonstrated the transcription of non-protein-coding centromere repeat RrS1 on lampbrush chromosomes of both *P. ridibundus* and *P. lessonae* as well as transcription of telomere repeat that proceeds in direction from subltelomere region to the chromosomal end. Constructed cytological maps and comprehensive description of landmark structures allow to identify individual chromosomes in lampbrush karyotypes of both parental species from Eastern Ukraine. The complete working maps of lampbrush chromosomes represent a useful instrument for high-resolution FISH-mapping of genomic fragments. Moreover, the lampbrush chromosome maps of *P. ridibundus* and *P. lessonae* will be used for identification of genomes transmitted in female germ cells of di- and triploid hybridogenic frogs (*P. esculentus*) from the Seversky Donets river basin.

## Methods

### Samples studied

The European water frogs were sampled in the Kharkov region (Eastern Ukraine). *P. ridibundus* (N=8) individuals were collected from various localities of the Seversky Donets River basin, where they form common population systems together with hybrid frogs. *P. lessonae* (N=7) individuals were caught from the adjacent region, the Dnieper River basin in Krasnokutsk district, where they form population systems together with *P. ridibundus* and hybrid frogs. All manipulations with animals were carried out in accordance with relevant federal guidelines and institutional policies.

### DNA flow cytometry

The amount of DNA per nucleus was measured in all *P. ridibundus* and *P. lessonae* females by flow cytometry, which was performed by using a microscope-based flow fluorimeter with mercury arc lamp as a light source. Multichannel analyzer connected with a microcomputer allowed to get DNA histograms.

After using of anesthesia, the blood was taken from the femoral vein. Blood samples were mixed with 0.1% Triton X100, 20 μg/ml ethidium bromide and 15 mM MgCl_2_. Measurements were made after 4–6 h (at +4°C). To estimate genome size of specimens under study their samples were compared with reference standard samples of *Rana temporaria* (St. Petersburg region), and then additionally with samples of male domestic mouse (*Mus musculus*; spleenocytes, C57B1 line, 6.8 pg, according to Bianchi et al. [56]). Nuclear DNA content was converted from relative unit to histograms using a formula: DNA content = (samples mean peak)/ (reference standard peak) × (reference standard genome size) [57,58].

### Lampbrush chromosome isolation

Spread preparations of *P. ridibundus* and *P. lessonae* oocyte nucleus contents were made according to procedure described by Callan et al. [59] with modifications suggested by Gall et al. [60]. Oocytes of 0.5–1.5 mm in diameter were obtained from non-stimulated females by manual dissection of ovary fragments within the OR2 saline (82.5 mM NaCl, 2.5 mM KCl, 1 mM MgCl_2_, 1 mM CaCl_2_, 1mM Na_2_HPO_4_, 5 mM HEPES (4-(2-hydroxyethyl)-1-piperazineethanesulfonic acid); pH 7.4). Nuclei were then microsurgically isolated from oocytes by jeweler forceps in the isolation medium “5:1” (83 mM KCl, 17 mM NaCl, 6.5 mM Na_2_HPO_4_, 3.5 mM KH_2_PO_4_, 1mM MgCl_2_, 1 mM DTT (dithiothreitol); pH 7.0–7.2). Nuclear envelopes were removed in one-fourth strength “5:1” medium with the addition of 0.1% paraformaldehyde and 0.01% 1M MgCl_2_ in a chambers attached to a specimen slide. All microsurgical procedures were performed under the observation at Leica MZ16 stereomicroscope. Then slide preparations of oocyte nuclei contents were centrifuged for 30 min at +4°C, 4000 rpm. After a brief fixation (for 30 min) in 2% paraformaldehyde in 1× phosphate buffered saline (PBS), preparations were post-fixed in 70% ethanol overnight (at +4°C). Preparations were not dried before immunostaining. Lampbrush chromosome maps were constructed as described in [18]. All marker structures on lampbrush chromosomes were assorted according to classification suggested by Callan [18].

### Preparation of mitotic and meiotic metaphase chromosomes

Mitotic and meiotic metaphase chromosomes were obtained from intestine and testes. Each individual was injected with 0.2–0.5 ml of a 0.3% solution of colchicine (48 hr prior to biopsy for intestinal tissue, 24 hr for testicular tissue). Tissue fragments were incubated in hypotonical solution for 20 minutes, then for 20 minutes in 45% acetic acid, and kept in 3:l ethanol-glacial acetic acid until slide preparation. The cell suspension was resuspended onto specimen slides. The slides were dried and stored at −20°C before use.

### Immunofluorescent staining of germinal vesicle spreads

For immunostaining of frog oocyte nucleus content preparations we used the following mouse monoclonal antibodies (mAb) and rabbit polyclonal antibodies (pAb): mAb No-185 against No38 protein [61], mAb No114 against Nopp140 protein [62], mAb 17с12 against fibrillarin [63], mAb 38F3 against fibrillarin (Santa Cruz Biotechnology), mAb K121 against 2,2,7-trimethyl guanosine cap (Santa Cruz Biotechnology), mAb Y12 against symmetrical dimethylarginine [64] and pAb R288 against С terminal domain of coilin [65]. Lampbrush chromosome spreads were incubated for 5 minutes in 70%, 50%, 30% ethanol, and in PBS with 0.01% Tween-20 and then were blocked in PBS containing 1% blocking reagent (Roche) for 1 h at RT, then incubated with primary antibody (dilutions as recommended by authors or manufacturers) for 1 h at RT. Slides were washed in PBS, 0.05% Tween-20 and incubated in corresponding secondary antibody or combination of antibodies (Cy3-conjugated goat anti-rabbit IgG (Jackson ImmunoResearch Laboratories), Alexa-488-conjugated goat anti-mouse IgG, Cy3-conjugated goat anti-mouse IgG and IgM (Molecular Probes)) for 1 h at RT. Slides were washed in PBS, 0.05% Tween-20 and mounted in DABCO antifade solution containing 1 mg/ml DAPI.

### Fluorescence *in situ* hybridization

A PCR product amplified from *P. lessonae* genomic DNA with following primers specific to RrS1 highly repetitive centromeric sequence [27] was used as a probe for fluorescence *in situ* hybridization (FISH):

F 5^′^-AAGCCGATTTTAGACAAGATTGC-3^′^;

R 5^′^-GGCCTTTGGTTACCAAATGC-3^′^

The probe was labelled with biotin-16-dUTP (Roche) by PCR with the same primers at the standard conditions. The labelled probes were dissolved to a final concentration of 10–50 ng/μl in a hybridization buffer (50% formamide, 2× SSC (3 M sodium chloride and 300 mM trisodium citrate), 10% dextran sulphate) with a 50-fold excess of salmon sperm DNA. In case of hybridization to lampbrush chromosomes, three variants of FISH were carried out: (1) DNA/DNA hybridization, with pre-treatment with RNase A; (2) DNA/(DNA+RNA) hybridization and (3) DNA/RNA hybridization, without RNase A treatment. In the first two variants, lampbrush chromosomes were denatured at 81.5°C for 5 min; in the third one chromosomal DNA was not denatured. Then slides were incubated with probe in hybridization buffer overnight at 37°С. After hybridization, slides were washed three times in 0.2 × SSC at 60°C and once in 2 × SSC at 42°C. Biotin was detected by avidin conjugated with Cy3 (Jackson ImmunoResearch Laboratories). All preparations after FISH were mounted in antifade solution containing 1 mg/ml DAPI.

In case of FISH to metaphase chromosomes, chromosome preparations were pre-treated with RNase A (100–200 μg/ml), pepsin (0.01% in 0.01 N HCl) and then post-fixed in formaldehyde (1% in PBS, 50 mM MgCl_2_). DNA/DNA hybridization was performed as described above.

DNA/RNA and DNA/(DNA+RNA) FISH with telomeric probe was preformed on lampbrush chromosomes at softer conditions as described by Solovei and co-authors [29]. Biotin conjugated telomeric single-stranded oligonucleotide probes (TAACCC)_5_ and (TTAGGG)_5_ were used for hybridization. The hybridization mixture contained 40% formamide, 2.4 × SSC, and 12% dextran sulphate, 5 ng/μl labeled probe and 10–50-fold excess of tRNA. For DNA/RNA hybridization chromosomes were not denatured. Hybridization was performed at room temperature for 12–18 h. After hybridization, slides were washed three times in 2 × SSC at 42°C. Biotin was detected by avidin conjugated with Cy3 (Jackson ImmunoResearch Laboratories). Chromosomes were counterstained with 1 mg/ml DAPI.

### Wide-field microscopy

Preparations of oocyte nuclei contents were examined using Leica fluorescence microscope DM4000 equipped with a monochrome digital camera DFC350 FX and appropriate filter cubes (Leica Wetzlar GmbH, Germany). Images were taken with 40×/1 and 100×/1.30 oil immersion objectives at RT. Leica CW 4000 FISH software was used for acquisition and processing the multicolor images.

## Abbreviations

DAPI: 4',6-diamidino-2-phenylindole; FISH: Fluorescence *in situ* hybridization; LBC: Lampbrush chromosome; LL2R: “Lumpy loop” 2 repeat; mAb: Monoclonal antibodies; NOR: Nucleolus organizer region; pAb: Polyclonal antibodies; PBS: Phosphate buffered saline; PCR: Polymerase chain reaction; RNP: Ribonucleoprotein; RrS1: *Rana ridibunda* sequence 1; snRNA: Small nuclear RNA; SSC: Saline-sodium citrate buffer; TERRA: Noncoding telomeric repeat-containing RNA; TMG cap: 2,2,7-trimethylguanosine cap.

## Competing interests

The authors declare that they have no competing interests.

## Authors’ contributions

DD and DS collected the samples. DD carried out the lampbrush chromosome isolation, performed immunofluorescent assays and FISH experiments, constructed the cytological maps and drafted the manuscript. AK analyzed the micrographs, verified the cytological maps, participated in the design of the study and its coordination and revised the manuscript. JR performed the DNA flow cytometry experiments. SL prepared metaphase chromosomes and participated in species identification. GM participated in lampbrush chromosome isolation. LB helped to discuss the obtained results. AS participated in the design of the study. All authors read and approved the final version of the manuscript.

## Supplementary Material

Additional file 1: Figure S1 Full set of lampbrush chromosomes from *P. ridibundus* oocytes. Immunofluorescent staining with antibodies K121 against TMG-cap of snRNAs reveals enriched marker loops. Chromosomes are counterstained with DAPI. Corresponding phase contrast micrographs are shown at Figure 1. Nu – extrachromosomal and chromosome associated nucleoli. Arrows indicate the most conspicuous marker loops. Scale bar = 50 μm. Click here for file

Additional file 2: Figure S2 Construction of cytological lampbrush chromosome map on example of *P. ridibundus* lampbrush chromosome H. **b**. Morphology of lampbrush chromosome H. Giant fusing loops, associated nucleoli and two pairs of marker loops are the most conspicuous marker structures. Dotted lines indicate two marker structures on lampbrush chromosomes, arrowheads show centromeres. Phase contrast micrograph. **a**. Plotting marker structures on the working chromosome map according to their relative position on lampbrush chromosome. Nu – extrachromosomal and chromosome associated nucleoli. Scale bars = 10 μm. Click here for file

Additional file 3: Figure S3 Comparison of *P. ridibundus* lampbrush chromosome С (**a, b**), D (**e, f**), G (**i, j**) and *P. lessonae* lampbrush chromosome D (**c, d**), C (**g, h**) and G (**k, l**). Phase contrast micrographs (**a, c, e, g, i, k**) and immunofluorescent staining with antibodies against TMG-cap of snRNAs (**b, d, f, h, j, l**). Chromosomes are counterstained with DAPI. Arrows indicate the most conspicuous marker structures on lampbrush chromosomes, arrowheads show centromeres. Nu – extra-chromosomal nucleoli. Scale bars = 10 μm. Click here for file

Additional file 4: Figure S4 Chromosome H of *P. lessonae.* Phase contrast micrograph (**a**), immunofluorescent staining with antibodies against TMG-cap of snRNAs (**b**), coilin (**c**) and FISH with (TAACCC)_5_-biotin oligonucleotide (**d**). Chromosomes are counterstained with DAPI. Arrows indicate lumpy and long marker loops in long arm of chromosome H of *P. lessonae* (**a, b**). Terminal and interstitial blocks of (TTAGGG)_n_ repeat (indicated by arrows) in *P. lessonae* lampbrush chromosome H (**d**). Arrows show coilin-positive granules in telomere regions and in interstitial sites corresponding to chromomeres containing (TTAGGG)-repeat (**c**). Arrowheads show centromeres. Nu – extrachromosomal nucleoli. Scale bars = 10 μm. Click here for file

Additional file 5: Figure S5 RrS1 centromere repeat mapping in metaphase chromosome preparations of both parental frog species from the Eastern Ukraine. FISH with RrS1 repeat in metaphase chromosomes of *P. ridibundus* (**a**) and *P. lessonae* (**b**). Arrows indicate clusters of RrS1 repeat in metaphase chromosomes of *P. ridibundus* and *P. lessonae*. Chromosomes are counterstained with DAPI. Scale bars = 10 μm. Click here for file

Additional file 6: Figure S6 RrS1 centromere repeat mapping in lampbrush chromosomes of *P. lessonae*. **a**. FISH of RrS1 repeat to lampbrush chromosomes of *P. lessonae*. Clusters of RrS1 repeat of various size localize in centromere regions (shown by arrowheads) of all chromosomes. Asterisks indicate enlarged fragments of lampbrush chromosomes. Scale bar = 50 μm. **b**, **c**, **d**. Fragments of chromosomes with conspicuous cluster of RrS1 repeat in a centromere region. Nu – extrachromosomal nucleoli. Chromosomes are counterstained with DAPI. Scale bars = 10 μm. Click here for file
