# Supplementary figures and images for: Cytological maps of lampbrush chromosomes of European water frogs (Pelophylax esculentus complex) from the Eastern Ukraine
Source: BMC Genet. 2013 Apr 16;14:26. doi: 10.1186/1471-2156-14-26 (PMC3648425; doi:10.1186/1471-2156-14-26)

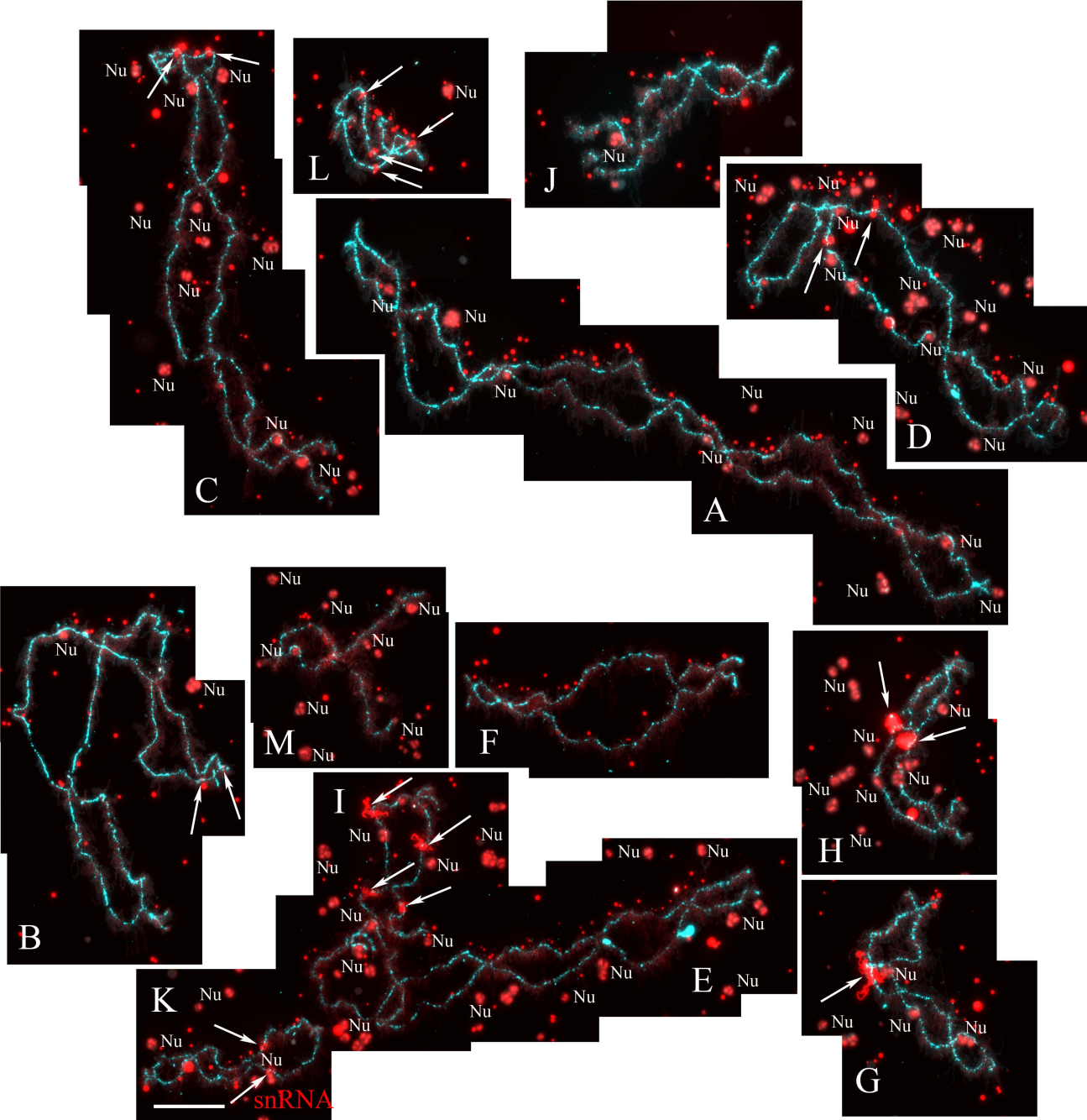

Supplement: Additional file 1: Figure S1 — Full set of lampbrush chromosomes from P. ridibundus oocytes. Immunofluorescent staining with antibodies K121 against TMG-cap of snRNAs reveals enriched marker loops. Chromosomes are counterstained with DAPI. Corresponding phase contrast micrographs are shown at Figure 1. Nu – extrachromosomal and chromosome associated nucleoli. Arrows indicate the most conspicuous marker loops. Scale bar = 50 μm. [file 1471-2156-14-26-S1.pdf]

a

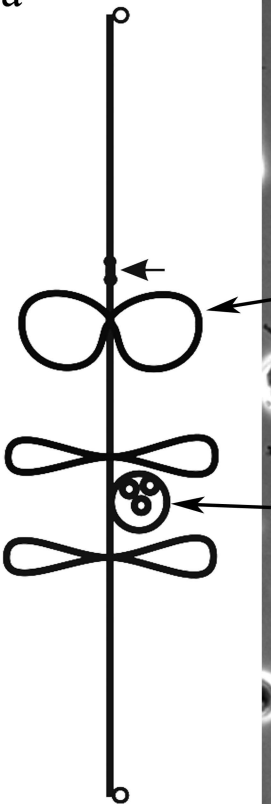

b

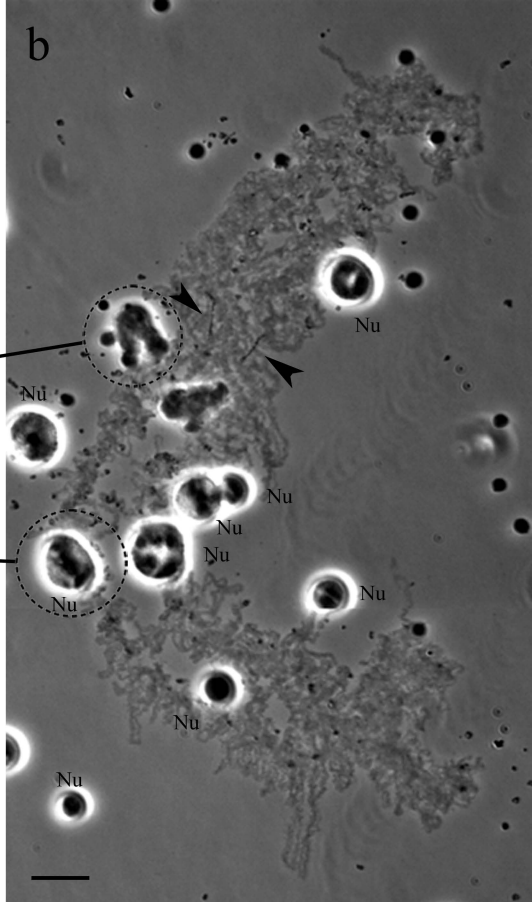

Supplement: Additional file 2: Figure S2 — Construction of cytological lampbrush chromosome map on example of P. ridibundus lampbrush chromosome H. b. Morphology of lampbrush chromosome H. Giant fusing loops, associated nucleoli and two pairs of marker loops are the most conspicuous marker structures. Dotted lines indicate two marker structures on lampbrush chromosomes, arrowheads show centromeres. Phase contrast micrograph. a. Plotting marker structures on the working chromosome map according to their relative position on lampbrush chromosome. Nu – extrachromosomal and chromosome associated nucleoli. Scale bars = 10 μm. [file 1471-2156-14-26-S2.pdf]

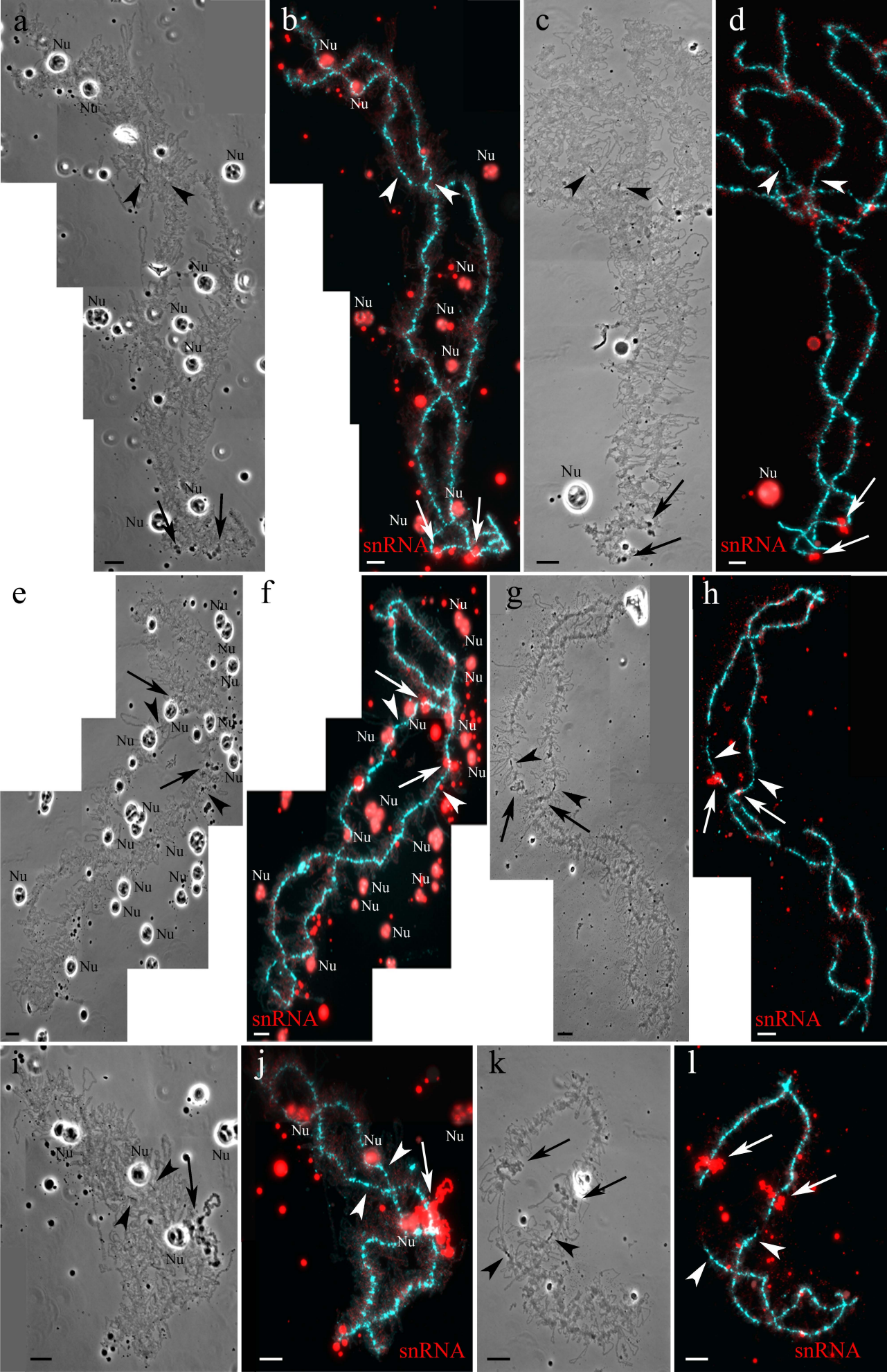

Supplement: Additional file 3: Figure S3 — Comparison of P. ridibundus lampbrush chromosome С (a, b), D (e, f), G (i, j) and P. lessonae lampbrush chromosome D (c, d), C (g, h) and G (k, l). Phase contrast micrographs (a, c, e, g, i, k) and immunofluorescent staining with antibodies against TMG-cap of snRNAs (b, d, f, h, j, l). Chromosomes are counterstained with DAPI. Arrows indicate the most conspicuous marker structures on lampbrush chromosomes, arrowheads show centromeres. Nu – extra-chromosomal nucleoli. Scale bars = 10 μm. [file 1471-2156-14-26-S3.pdf]

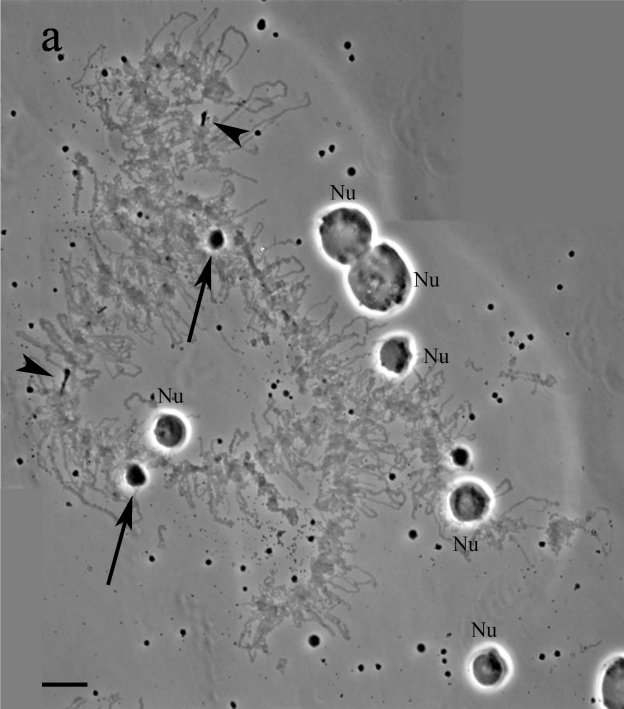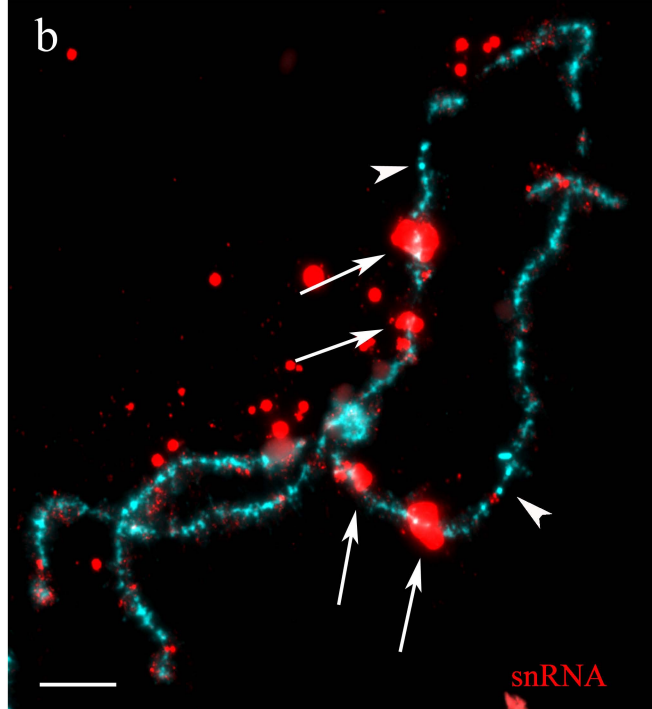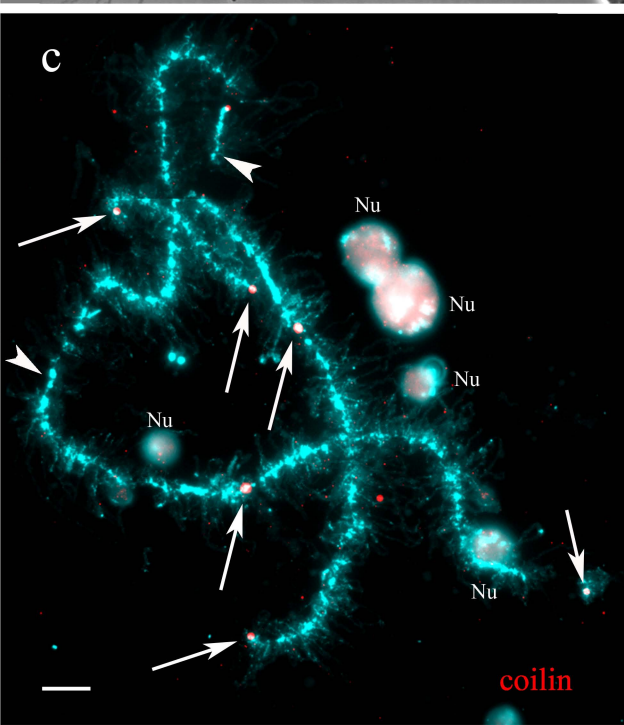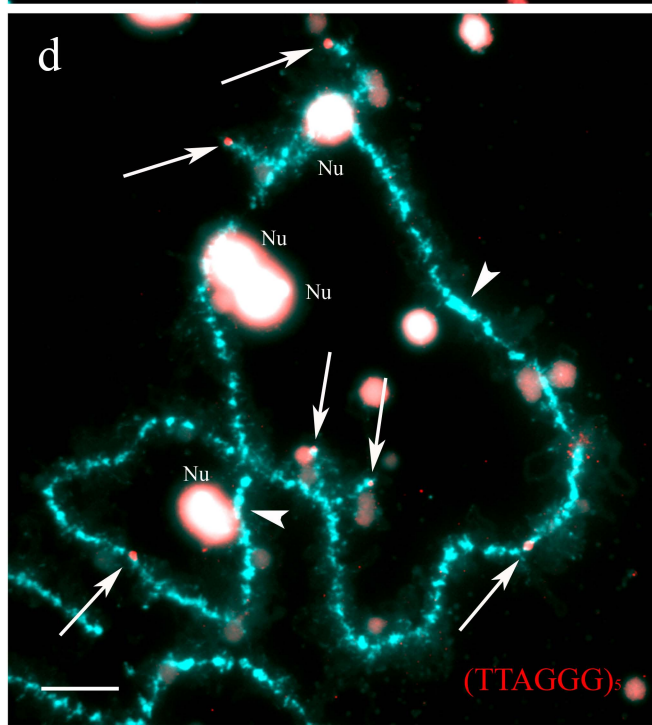

Supplement: Additional file 4: Figure S4 — Chromosome H of P. lessonae. Phase contrast micrograph (a), immunofluorescent staining with antibodies against TMG-cap of snRNAs (b), coilin (c) and FISH with (TAACCC)5-biotin oligonucleotide (d). Chromosomes are counterstained with DAPI. Arrows indicate lumpy and long marker loops in long arm of chromosome H of P. lessonae (a, b). Terminal and interstitial blocks of (TTAGGG)n repeat (indicated by arrows) in P. lessonae lampbrush chromosome H (d). Arrows show coilin-positive granules in telomere regions and in interstitial sites corresponding to chromomeres containing (TTAGGG)-repeat (c). Arrowheads show centromeres. Nu – extrachromosomal nucleoli. Scale bars = 10 μm. [file 1471-2156-14-26-S4.pdf]

a

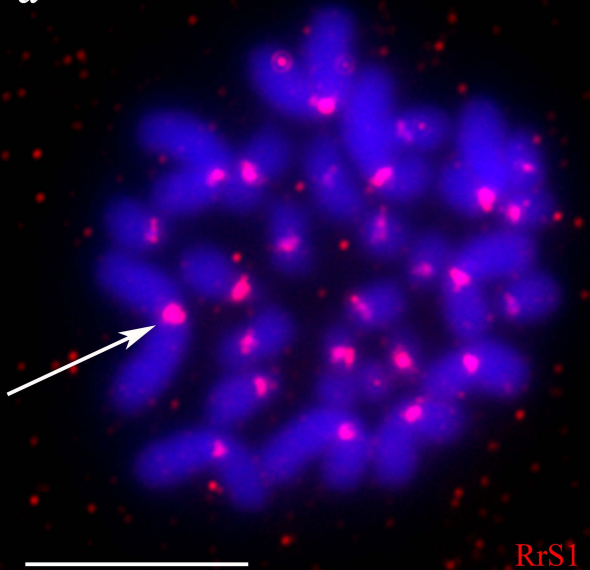

RrS1

b

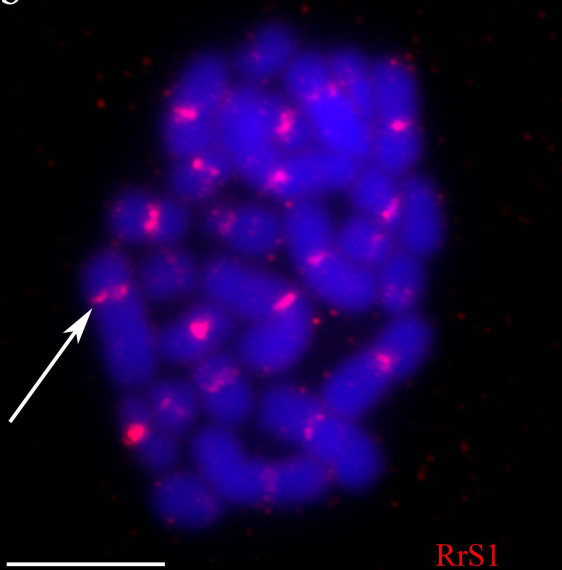

RrS1

Supplement: Additional file 5: Figure S5 — RrS1 centromere repeat mapping in metaphase chromosome preparations of both parental frog species from the Eastern Ukraine. FISH with RrS1 repeat in metaphase chromosomes of P. ridibundus (a) and P. lessonae (b). Arrows indicate clusters of RrS1 repeat in metaphase chromosomes of P. ridibundus and P. lessonae. Chromosomes are counterstained with DAPI. Scale bars = 10 μm. [file 1471-2156-14-26-S5.pdf]
