# Supplementary material for: Cytological maps of lampbrush chromosomes of European water frogs (Pelophylax esculentus complex) from the Eastern Ukraine
Source: BMC Genet. 2013 Apr 16;14:26. doi: 10.1186/1471-2156-14-26 (PMC3648425; doi:10.1186/1471-2156-14-26)
Supplement: Additional file 6: Figure S6 — RrS1 centromere repeat mapping in lampbrush chromosomes of P. lessonae. a. FISH of RrS1 repeat to lampbrush chromosomes of P. lessonae. Clusters of RrS1 repeat of various size localize in centromere regions (shown by arrowheads) of all chromosomes. Asterisks indicate enlarged fragments of lampbrush chromosomes. Scale bar = 50 μm. b, c, d. Fragments of chromosomes with conspicuous cluster of RrS1 repeat in a centromere region. Nu – extrachromosomal nucleoli. Chromosomes are counterstained with DAPI. Scale bars = 10 μm. [file 1471-2156-14-26-S6.pdf]

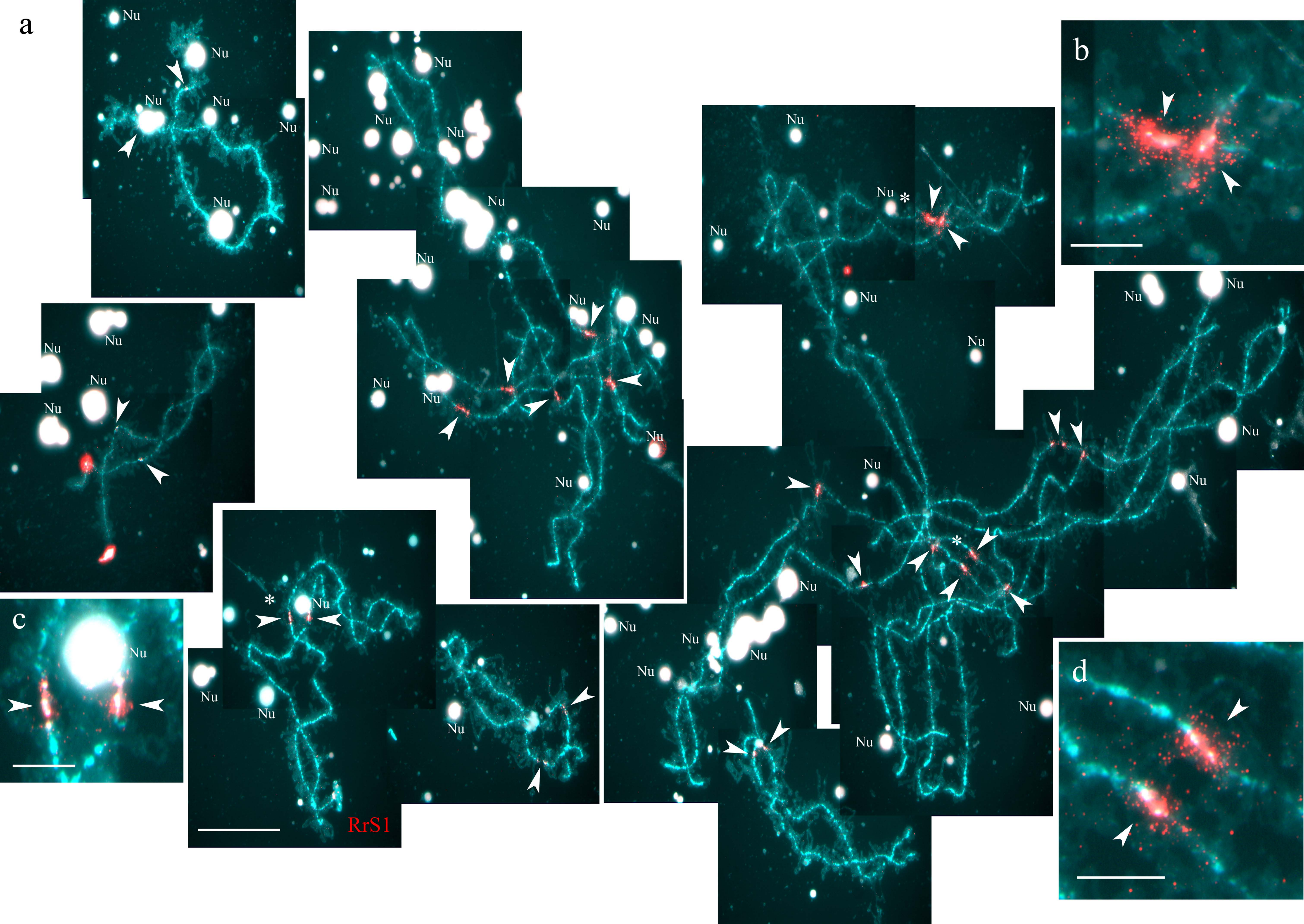

a
b
c
d
Nu
*
RrS1
